# Supplementary material for: A New Functional Screening Platform Identifies Colistin Sulfate as an Enhancer of Natural Killer Cell Cytotoxicity
Source: Cancers (Basel). 2022 Jun 8;14(12):2832. doi: 10.3390/cancers14122832 (PMC9221353; doi:10.3390/cancers14122832)
Supplement: Supplementary file 1 [file cancers-14-02832-s001.zip › cancers-1767069-supplementary.pdf]

# Contents

**Supplemental Table 1. Fold-change of NK92 and K562-NL cells treated with compounds from the Prestwick Chemical Library drug screening.** ........ 1

**Supplemental Table 2. Compounds of the Prestwick Chemical Library excluded from analysis.** ... 53

**Supplemental Table 3. Z' factor for individual assay plates from the screening of the Prestwick Chemical Library.** ........ 54

**Supplemental Table 1. Fold-change of NK92 and K562-NL cells treated with compounds from the Prestwick Chemical Library drug screening.** Fold-change of luminescent values of all wells containing compounds compared DMSO control wells in the same plate. Each compound was evaluated in singlet over 2 biological replicates. [a]Plates 5 & 7: fold-change of one replicate.

| Prestw number | Plate # / Well position | Chemical name | Target alone | 1:1 E:T |
|---|---|---|---|---|
| ***Plate 1*** | | | | |
| **Prestw-1** | 01A02 | Azaguanine-8 | 0.84 | 1.16 |
| **Prestw-2** | 01A03 | Allantoin | 0.86 | 1.15 |
| **Prestw-3** | 01A04 | Acetazolamide | 0.87 | 1.13 |
| **Prestw-4** | 01A05 | Metformin hydrochloride | 0.86 | 1.16 |
| **Prestw-5** | 01A06 | Atracurium besylate | 0.83 | 1.15 |
| **Prestw-6** | 01A07 | Isoflupredone acetate | 0.76 | 1.19 |
| **Prestw-7** | 01A08 | Amiloride hydrochloride dihydrate | 0.89 | 1.20 |
| **Prestw-8** | 01A09 | Amprolium hydrochloride | 0.85 | 1.19 |
| **Prestw-9** | 01A10 | Hydrochlorothiazide | 0.81 | 1.20 |
| **Prestw-10** | 01A11 | Sulfaguanidine | 0.79 | 1.16 |
| **Prestw-11** | 01B02 | Meticrane | 0.86 | 1.05 |
| **Prestw-12** | 01B03 | Benzonatate | 0.81 | 1.16 |
| **Prestw-13** | 01B04 | Hydroflumethiazide | 0.92 | 1.16 |
| **Prestw-14** | 01B05 | Sulfacetamide sodic hydrate | 0.83 | 1.09 |
| **Prestw-15** | 01B06 | Heptaminol hydrochloride | 0.87 | 1.06 |
| **Prestw-16** | 01B07 | Sulfathiazole | 0.88 | 1.17 |
| **Prestw-17** | 01B08 | Levodopa | 0.86 | 1.12 |
| **Prestw-18** | 01B09 | Idoxuridine | 0.89 | 1.17 |
| **Prestw-19** | 01B10 | Captopril | 0.84 | 1.11 |
| **Prestw-20** | 01B11 | Minoxidil | 0.88 | 1.10 |

| | | | | |
|---|---|---|---|---|
| **Prestw-21** | 01C02 | Sulfaphenazole | 0.91 | 1.10 |
| **Prestw-22** | 01C03 | Panthenol (D) | 0.97 | 1.15 |
| **Prestw-23** | 01C04 | Sulfadiazine | 0.93 | 1.06 |
| **Prestw-24** | 01C05 | Norethynodrel | 0.98 | 1.08 |
| **Prestw-25** | 01C06 | Thiamphenicol | 0.91 | 1.11 |
| **Prestw-26** | 01C07 | Cimetidine | 0.90 | 1.10 |
| **Prestw-27** | 01C08 | Doxylamine succinate | 0.93 | 1.08 |
| **Prestw-28** | 01C09 | Ethambutol dihydrochloride | 0.81 | 1.12 |
| **Prestw-29** | 01C10 | Antipyrine | 0.80 | 1.00 |
| **Prestw-30** | 01C11 | Antipyrine, 4-hydroxy | 0.83 | 1.10 |
| **Prestw-31** | 01D02 | Chloramphenicol | 0.89 | 1.04 |
| **Prestw-32** | 01D03 | Epirizole | 0.88 | 1.03 |
| **Prestw-33** | 01D04 | Diprophylline | 0.85 | 1.04 |
| **Prestw-34** | 01D05 | Triamterene | 0.92 | 1.08 |
| **Prestw-35** | 01D06 | Dapsone | 0.94 | 1.08 |
| **Prestw-36** | 01D07 | Troleandomycin | 0.91 | 1.15 |
| **Prestw-37** | 01D08 | Pyrimethamine | 0.82 | 1.06 |
| **Prestw-38** | 01D09 | Hexamethonium dibromide dihydrate | 0.89 | 1.13 |
| **Prestw-39** | 01D10 | Diflunisal | 0.79 | 1.06 |
| **Prestw-40** | 01D11 | Niclosamide | 0.81 | 1.18 |
| **Prestw-41** | 01E02 | Procaine hydrochloride | 0.92 | 1.07 |
| **Prestw-42** | 01E03 | Moxisylyte hydrochoride | 0.94 | 1.10 |
| **Prestw-43** | 01E04 | Betazole hydrochloride | 0.95 | 1.06 |
| **Prestw-44** | 01E05 | Isoxicam | 0.91 | 1.00 |
| **Prestw-45** | 01E06 | Naproxen | 0.91 | 1.05 |
| **Prestw-46** | 01E07 | Naphazoline hydrochloride | 0.92 | 1.08 |
| **Prestw-47** | 01E08 | Ticlopidine hydrochloride | 0.88 | 1.12 |
| **Prestw-48** | 01E09 | Dicyclomine hydrochloride | 0.89 | 1.05 |
| **Prestw-49** | 01E10 | Amyleine hydrochloride | 0.84 | 1.04 |
| **Prestw-50** | 01E11 | Lidocaïne hydrochloride | 0.92 | 1.04 |
| **Prestw-51** | 01F02 | Trichlorfon | 0.93 | 0.99 |
| **Prestw-52** | 01F03 | Carbamazepine | 0.95 | 1.02 |
| **Prestw-53** | 01F04 | Triflupromazine hydrochloride | 0.94 | 0.96 |
| **Prestw-54** | 01F05 | Mefenamic acid | 0.91 | 1.05 |
| **Prestw-55** | 01F06 | Acetohexamide | 0.95 | 1.06 |
| **Prestw-56** | 01F07 | Sulpiride | 0.93 | 1.02 |
| **Prestw-57** | 01F08 | Benoxinate hydrochloride | 0.85 | 1.00 |
| **Prestw-58** | 01F09 | Oxethazaine | 0.82 | 1.01 |
| **Prestw-59** | 01F10 | Pheniramine maleate | 0.80 | 1.13 |
| **Prestw-60** | 01F11 | Tolazoline hydrochloride | 0.81 | 1.02 |
| **Prestw-61** | 01G02 | Morantel tartrate | 0.87 | 1.00 |
| **Prestw-62** | 01G03 | Homatropine hydrobromide (R,S) | 0.89 | 0.99 |
| **Prestw-63** | 01G04 | Nifedipine | 0.79 | 1.03 |

| | | | | |
|---|---|---|---|---|
| **Prestw-64** | 01G05 | Chlorpromazine hydrochloride | 0.91 | 1.01 |
| **Prestw-65** | 01G06 | Diphenhydramine hydrochloride | 0.77 | 0.96 |
| **Prestw-66** | 01G07 | Minaprine dihydrochloride | 0.83 | 1.03 |
| **Prestw-67** | 01G08 | Miconazole | 0.85 | 1.06 |
| **Prestw-68** | 01G09 | Isoxsuprine hydrochloride | 0.84 | 1.00 |
| **Prestw-69** | 01G10 | Acebutolol hydrochloride | 0.85 | 1.02 |
| **Prestw-70** | 01G11 | Tolnaftate | 0.85 | 1.06 |
| **Prestw-71** | 01H02 | Todralazine hydrochloride | 0.82 | 1.02 |
| **Prestw-72** | 01H03 | Imipramine hydrochloride | 0.81 | 0.91 |
| **Prestw-73** | 01H04 | Sulindac | 0.83 | 0.99 |
| **Prestw-74** | 01H05 | Amitryptiline hydrochloride | 0.90 | 0.91 |
| **Prestw-75** | 01H06 | Adiphenine hydrochloride | 0.87 | 1.01 |
| **Prestw-76** | 01H07 | Dibucaine | 0.87 | 0.99 |
| **Prestw-77** | 01H08 | Prednisone | 0.85 | 1.02 |
| **Prestw-78** | 01H09 | Thioridazine hydrochloride | 0.82 | 0.98 |
| **Prestw-79** | 01H10 | Diphemanil methylsulfate | 0.72 | 0.99 |
| **Prestw-80** | 01H11 | Trimethobenzamide hydrochloride | 0.84 | 1.06 |
| ***Plate 2*** | | | | |
| **Prestw-81** | 02A02 | Metronidazole | 0.89 | 1.16 |
| **Prestw-1424** | 02A03 | Fulvestrant | 0.97 | 1.16 |
| **Prestw-83** | 02A04 | Edrophonium chloride | 0.92 | 1.24 |
| **Prestw-84** | 02A05 | Moroxidine hydrochloride | 0.83 | 1.13 |
| **Prestw-85** | 02A06 | Baclofen (R,S) | 0.94 | 1.13 |
| **Prestw-86** | 02A07 | Acyclovir | 0.95 | 1.17 |
| **Prestw-87** | 02A08 | Diazoxide | 0.91 | 1.19 |
| **Prestw-88** | 02A09 | Amidopyrine | 0.93 | 1.12 |
| **Prestw-1179** | 02A10 | Busulfan | 0.90 | 1.20 |
| **Prestw-90** | 02A11 | Pindolol | 0.91 | 1.19 |
| **Prestw-91** | 02B02 | Khellin | 0.86 | 1.03 |
| **Prestw-92** | 02B03 | Zimelidine dihydrochloride monohydrate | 0.92 | 1.08 |
| **Prestw-93** | 02B04 | Azacyclonol | 0.86 | 1.03 |
| **Prestw-94** | 02B05 | Azathioprine | 0.89 | 1.07 |
| **Prestw-95** | 02B06 | Lynestrenol | 0.90 | 1.10 |
| **Prestw-96** | 02B07 | Guanabenz acetate | 0.95 | 1.19 |
| **Prestw-97** | 02B08 | Disulfiram | 0.95 | 0.87 |
| **Prestw-98** | 02B09 | Acetylsalicylsalicylic acid | 0.93 | 1.23 |
| **Prestw-99** | 02B10 | Mianserine hydrochloride | 0.96 | 1.08 |
| **Prestw-100** | 02B11 | Nocodazole | 0.99 | 1.25 |

| | | | | |
|---|---|---|---|---|
| **Prestw-101** | 02C02 | R(-) Apomorphine hydrochloride hemihydrate | 0.89 | 1.10 |
| **Prestw-102** | 02C03 | Amoxapine | 0.87 | 0.86 |
| **Prestw-103** | 02C04 | Cyproheptadine hydrochloride | 0.84 | 1.09 |
| **Prestw-104** | 02C05 | Famotidine | 0.88 | 1.16 |
| **Prestw-105** | 02C06 | Danazol | 0.98 | 1.07 |
| **Prestw-106** | 02C07 | Nicorandil | 0.93 | 1.14 |
| **Prestw-1314** | 02C08 | Pioglitazone | 1.02 | 1.16 |
| **Prestw-108** | 02C09 | Nomifensine maleate | 0.89 | 1.04 |
| **Prestw-109** | 02C10 | Dizocilpine maleate | 0.86 | 1.04 |
| **Prestw-1192** | 02C11 | Oxandrolone | 0.83 | 1.16 |
| **Prestw-111** | 02D02 | Naloxone hydrochloride | 0.94 | 1.08 |
| **Prestw-112** | 02D03 | Metolazone | 0.80 | 1.01 |
| **Prestw-113** | 02D04 | Ciprofloxacin hydrochloride | 0.93 | 1.02 |
| **Prestw-114** | 02D05 | Ampicillin trihydrate | 0.91 | 1.06 |
| **Prestw-115** | 02D06 | Haloperidol | 0.95 | 1.06 |
| **Prestw-116** | 02D07 | Naltrexone hydrochloride dihydrate | 0.90 | 1.06 |
| **Prestw-117** | 02D08 | Chlorpheniramine maleate | 0.90 | 1.00 |
| **Prestw-118** | 02D09 | Nalbuphine hydrochloride | 0.85 | 1.05 |
| **Prestw-119** | 02D10 | Picotamide monohydrate | 0.83 | 1.13 |
| **Prestw-120** | 02D11 | Triamcinolone | 0.81 | 1.14 |
| **Prestw-121** | 02E02 | Bromocryptine mesylate | 0.85 | 1.24 |
| **Prestw-1471** | 02E03 | Amfepramone hydrochloride | 0.93 | 1.08 |
| **Prestw-123** | 02E04 | Dehydrocholic acid | 0.93 | 1.15 |

| | | | | |
|---|---|---|---|---|
| **Prestw-1184** | 02E05 | Tioconazole | 0.94 | 0.98 |
| **Prestw-125** | 02E06 | Perphenazine | 0.98 | 0.45 |
| **Prestw-126** | 02E07 | Mefloquine hydrochloride | 0.95 | 1.03 |
| **Prestw-127** | 02E08 | Isoconazole | 0.88 | 1.04 |
| **Prestw-128** | 02E09 | Spironolactone | 0.86 | 1.11 |
| **Prestw-129** | 02E10 | Pirenzepine dihydrochloride | 0.88 | 1.09 |
| **Prestw-130** | 02E11 | Dexamethasone acetate | 0.88 | 1.13 |
| **Prestw-131** | 02F02 | Glipizide | 0.86 | 1.07 |
| **Prestw-132** | 02F03 | Loxapine succinate | 0.78 | 0.90 |
| **Prestw-133** | 02F04 | Hydroxyzine dihydrochloride | 0.77 | 0.80 |
| **Prestw-134** | 02F05 | Diltiazem hydrochloride | 1.00 | 1.03 |
| **Prestw-135** | 02F06 | Methotrexate | 0.88 | 1.10 |
| **Prestw-136** | 02F07 | Astemizole | 0.94 | 0.77 |
| **Prestw-137** | 02F08 | Clindamycin hydrochloride | 0.92 | 1.08 |
| **Prestw-138** | 02F09 | Terfenadine | 1.10 | 0.86 |
| **Prestw-139** | 02F10 | Cefotaxime sodium salt | 0.93 | 1.11 |
| **Prestw-140** | 02F11 | Tetracycline hydrochloride | 0.84 | 1.14 |
| **Prestw-141** | 02G02 | Verapamil hydrochloride | 0.87 | 0.99 |
| **Prestw-142** | 02G03 | Dipyridamole | 0.93 | 1.03 |
| **Prestw-143** | 02G04 | Chlorhexidine | 1.36 | 0.41 |
| **Prestw-144** | 02G05 | Loperamide hydrochloride | 0.93 | 0.83 |
| **Prestw-145** | 02G06 | Chlortetracycline hydrochloride | 0.91 | 1.03 |
| **Prestw-146** | 02G07 | Tamoxifen citrate | 0.87 | 1.00 |

| | | | | |
|---|---|---|---|---|
| **Prestw-147** | 02G08 | Nicergoline | 0.90 | 1.03 |
| **Prestw-148** | 02G09 | Canrenoic acid potassium salt | 0.87 | 1.01 |
| **Prestw-149** | 02G10 | Thioproperazine dimesylate | 0.86 | 0.77 |
| **Prestw-150** | 02G11 | Dihydroergotamine tartrate | 0.89 | 0.82 |
| **Prestw-151** | 02H02 | Erythromycin | 0.76 | 0.95 |
| **Prestw-1474** | 02H03 | Chloroxine | 0.84 | 0.97 |
| **Prestw-153** | 02H04 | Didanosine | 0.87 | 0.97 |
| **Prestw-154** | 02H05 | Josamycin | 0.87 | 0.97 |
| **Prestw-155** | 02H06 | Paclitaxel | 0.82 | 0.94 |
| **Prestw-156** | 02H07 | Ivermectin | 0.79 | 1.05 |
| **Prestw-157** | 02H08 | Gallamine triethiodide | 0.79 | 0.95 |
| **Prestw-158** | 02H09 | Neomycin sulfate | 0.87 | 1.02 |
| **Prestw-159** | 02H10 | Dihydrostreptomycin sulfate | 0.85 | 1.01 |
| **Prestw-160** | 02H11 | Gentamicine sulfate | 0.89 | 1.06 |
| ***Plate 3*** | | | | |
| **Prestw-161** | 03A02 | Isoniazid | 0.90 | 1.10 |
| **Prestw-162** | 03A03 | Pentylenetetrazole | 0.95 | 1.14 |
| **Prestw-163** | 03A04 | Chlorzoxazone | 0.89 | 1.09 |
| **Prestw-164** | 03A05 | Ornidazole | 0.86 | 1.17 |
| **Prestw-165** | 03A06 | Ethosuximide | 0.94 | 1.08 |
| **Prestw-166** | 03A07 | Mafenide hydrochloride | 0.97 | 1.22 |
| **Prestw-167** | 03A08 | Riluzole hydrochloride | 0.95 | 0.94 |
| **Prestw-168** | 03A09 | Nitrofurantoin | 0.91 | 1.11 |

| | | | | |
|---|---|---|---|---|
| **Prestw-169** | 03A10 | Hydralazine hydrochloride | 0.87 | 1.14 |
| **Prestw-170** | 03A11 | Phenelzine sulfate | 0.96 | 1.26 |
| **Prestw-171** | 03B02 | Tranexamic acid | 0.86 | 1.04 |
| **Prestw-172** | 03B03 | Etofylline | 1.01 | 1.18 |
| **Prestw-173** | 03B04 | Tranylcypromine hydrochloride | 0.90 | 1.07 |
| **Prestw-174** | 03B05 | Alverine citrate salt | 0.97 | 1.10 |
| **Prestw-175** | 03B06 | Aceclofenac | 0.92 | 1.07 |
| **Prestw-176** | 03B07 | Iproniazide phosphate | 0.88 | 1.14 |
| **Prestw-177** | 03B08 | Sulfamethoxazole | 0.84 | 1.02 |
| **Prestw-178** | 03B09 | Mephenesin | 0.90 | 1.07 |
| **Prestw-179** | 03B10 | Phenformin hydrochloride | 0.86 | 1.12 |
| **Prestw-180** | 03B11 | Flutamide | 0.96 | 0.96 |
| **Prestw-181** | 03C02 | Ampyrone | 0.93 | 1.05 |
| **Prestw-182** | 03C03 | Levamisole hydrochloride | 0.84 | 0.94 |
| **Prestw-183** | 03C04 | Pargyline hydrochloride | 0.83 | 1.03 |
| **Prestw-184** | 03C05 | Methocarbamol | 0.95 | 1.08 |
| **Prestw-185** | 03C06 | Aztreonam | 0.86 | 1.06 |
| **Prestw-186** | 03C07 | Cloxacillin sodium salt | 0.88 | 1.02 |
| **Prestw-187** | 03C08 | Catharanthine | 0.89 | 1.06 |
| **Prestw-188** | 03C09 | Pentolinium bitartrate | 0.85 | 1.02 |
| **Prestw-189** | 03C10 | Aminopurine, 6-benzyl | 0.69 | 0.85 |
| **Prestw-190** | 03C11 | Tolbutamide | 0.88 | 0.84 |
| **Prestw-191** | 03D02 | Midodrine hydrochloride | 0.87 | 0.96 |

| | | | | |
|---|---|---|---|---|
| **Prestw-192** | 03D03 | Thalidomide | 0.88 | 0.94 |
| **Prestw-193** | 03D04 | Oxolinic acid | 0.94 | 0.98 |
| **Prestw-194** | 03D05 | Nimesulide | 0.90 | 1.06 |
| **Prestw-195** | 03D06 | Hydrastinine hydrochloride | 0.85 | 0.98 |
| **Prestw-196** | 03D07 | Pentoxifylline | 0.93 | 0.97 |
| **Prestw-197** | 03D08 | Metaraminol bitartrate | 0.85 | 0.99 |
| **Prestw-198** | 03D09 | Salbutamol | 0.86 | 1.11 |
| **Prestw-199** | 03D10 | Prilocaine hydrochloride | 0.91 | 0.99 |
| **Prestw-200** | 03D11 | Camptothecine (S,+) | 0.88 | 0.87 |
| **Prestw-201** | 03E02 | Ranitidine hydrochloride | 0.90 | 1.11 |
| **Prestw-202** | 03E03 | Tiratricol, 3,3',5-triiodothyroacetic acid | 0.83 | 1.06 |
| **Prestw-203** | 03E04 | Flufenamic acid | 0.92 | 1.05 |
| **Prestw-204** | 03E05 | Flumequine | 0.98 | 1.09 |
| **Prestw-205** | 03E06 | Tolfenamic acid | 0.90 | 1.10 |
| **Prestw-206** | 03E07 | Meclofenamic acid sodium salt monohydrate | 0.85 | 1.06 |
| **Prestw-1181** | 03E08 | Tibolone | 1.04 | 1.02 |
| **Prestw-208** | 03E09 | Trimethoprim | 0.94 | 1.03 |
| **Prestw-209** | 03E10 | Metoclopramide monohydrochloride | 0.87 | 1.03 |
| **Prestw-210** | 03E11 | Fenbendazole | 0.90 | 0.90 |
| **Prestw-211** | 03F02 | Piroxicam | 0.92 | 0.94 |
| **Prestw-212** | 03F03 | Pyrantel tartrate | 0.86 | 0.93 |
| **Prestw-213** | 03F04 | Fenspiride hydrochloride | 0.93 | 0.97 |
| **Prestw-214** | 03F05 | Gemfibrozil | 0.87 | 0.95 |

| | | | | |
|---|---|---|---|---|
| **Prestw-215** | 03F06 | Mefexamide hydrochloride | 0.85 | 0.97 |
| **Prestw-216** | 03F07 | Tiapride hydrochloride | 0.92 | 1.05 |
| **Prestw-217** | 03F08 | Mebendazole | 0.91 | 1.03 |
| **Prestw-218** | 03F09 | Fenbufen | 0.86 | 1.03 |
| **Prestw-219** | 03F10 | Ketoprofen | 0.88 | 0.96 |
| **Prestw-220** | 03F11 | Indapamide | 0.88 | 1.01 |
| **Prestw-221** | 03G02 | Norfloxacin | 0.82 | 1.01 |
| **Prestw-222** | 03G03 | Antimycin A | 0.95 | 1.15 |
| **Prestw-223** | 03G04 | Xylometazoline hydrochloride | 0.87 | 1.00 |
| **Prestw-224** | 03G05 | Oxymetazoline hydrochloride | 0.89 | 1.02 |
| **Prestw-225** | 03G06 | Nifenazone | 0.87 | 0.95 |
| **Prestw-226** | 03G07 | Griseofulvin | 0.75 | 0.95 |
| **Prestw-227** | 03G08 | Clemizole hydrochloride | 0.88 | 1.02 |
| **Prestw-228** | 03G09 | Tropicamide | 0.86 | 0.83 |
| **Prestw-229** | 03G10 | Nefopam hydrochloride | 0.88 | 0.70 |
| **Prestw-230** | 03G11 | Phentolamine hydrochloride | 0.86 | 0.75 |
| **Prestw-231** | 03H02 | Etodolac | 0.92 | 1.07 |
| **Prestw-232** | 03H03 | Scopolamin-N-oxide hydrobromide | 0.95 | 0.97 |
| **Prestw-233** | 03H04 | Hyoscyamine (L) | 0.95 | 1.01 |
| **Prestw-234** | 03H05 | Chlorphensin carbamate | 0.92 | 0.99 |
| **Prestw-235** | 03H06 | Metampicillin sodium salt | 0.92 | 0.94 |
| **Prestw-236** | 03H07 | Dilazep dihydrochloride | 0.95 | 1.01 |
| **Prestw-237** | 03H08 | Ofloxacin | 0.97 | 1.05 |

| | | | | |
|---|---|---|---|---|
| **Prestw-238** | 03H09 | Lomefloxacin hydrochloride | 0.96 | 1.03 |
| **Prestw-239** | 03H10 | Orphenadrine hydrochloride | 0.95 | 0.91 |
| **Prestw-240** | 03H11 | Proglumide | 0.85 | 1.08 |
| ***Plate 4*** | | | | |
| **Prestw-241** | 04A02 | Mexiletine hydrochloride | 0.91 | 1.20 |
| **Prestw-242** | 04A03 | Flavoxate hydrochloride | 0.84 | 1.16 |
| **Prestw-243** | 04A04 | Bufexamac | 0.85 | 1.09 |
| **Prestw-244** | 04A05 | Glutethimide, para-amino | 0.96 | 1.22 |
| **Prestw-245** | 04A06 | Dropropizine (R,S) | 0.89 | 1.21 |
| **Prestw-246** | 04A07 | Pinacidil | 0.92 | 1.03 |
| **Prestw-247** | 04A08 | Albendazole | 0.92 | 1.02 |
| **Prestw-248** | 04A09 | Clonidine hydrochloride | 0.86 | 1.20 |
| **Prestw-249** | 04A10 | Bupropion hydrochloride | 0.86 | 1.29 |
| **Prestw-250** | 04A11 | Alprenolol hydrochloride | 0.90 | 1.18 |
| **Prestw-251** | 04B02 | Chlorothiazide | 0.82 | 0.95 |
| **Prestw-252** | 04B03 | Diphenidol hydrochloride | 0.78 | 1.10 |
| **Prestw-253** | 04B04 | Norethindrone | 0.80 | 1.13 |
| **Prestw-254** | 04B05 | Nortriptyline hydrochloride | 0.80 | 0.99 |
| **Prestw-255** | 04B06 | Niflumic acid | 0.82 | 1.17 |
| **Prestw-256** | 04B07 | Isotretinoin | 0.84 | 1.23 |
| **Prestw-257** | 04B08 | Retinoic acid | 0.89 | 1.24 |
| **Prestw-258** | 04B09 | Antazoline hydrochloride | 0.83 | 1.11 |
| **Prestw-259** | 04B10 | Ethacrynic acid | 0.89 | 1.20 |

| | | | | |
|---|---|---|---|---|
| **Prestw-260** | 04B11 | Praziquantel | 0.86 | 1.10 |
| **Prestw-261** | 04C02 | Ethisterone | 0.81 | 1.04 |
| **Prestw-262** | 04C03 | Triprolidine hydrochloride | 0.83 | 1.12 |
| **Prestw-263** | 04C04 | Doxepin hydrochloride | 0.86 | 0.86 |
| **Prestw-264** | 04C05 | Dyclonine hydrochloride | 0.76 | 1.01 |
| **Prestw-265** | 04C06 | Dimenhydrinate | 0.82 | 1.09 |
| **Prestw-266** | 04C07 | Disopyramide | 0.87 | 1.14 |
| **Prestw-267** | 04C08 | Clotrimazole | 0.92 | 1.06 |
| **Prestw-268** | 04C09 | Vinpocetine | 0.86 | 1.25 |
| **Prestw-269** | 04C10 | Clomipramine hydrochloride | 0.84 | 0.97 |
| **Prestw-270** | 04C11 | Fendiline hydrochloride | 0.89 | 0.91 |
| **Prestw-271** | 04D02 | Vincamine | 0.82 | 1.12 |
| **Prestw-272** | 04D03 | Indomethacin | 0.83 | 1.02 |
| **Prestw-273** | 04D04 | Cortisone | 0.78 | 1.11 |
| **Prestw-274** | 04D05 | Prednisolone | 0.98 | 1.16 |
| **Prestw-275** | 04D06 | Fenofibrate | 0.82 | 1.12 |
| **Prestw-276** | 04D07 | Bumetanide | 0.88 | 1.25 |
| **Prestw-277** | 04D08 | Labetalol hydrochloride | 0.84 | 1.09 |
| **Prestw-278** | 04D09 | Cinnarizine | 0.86 | 1.18 |
| **Prestw-279** | 04D10 | Methylprednisolone, 6-alpha | 0.80 | 1.26 |
| **Prestw-280** | 04D11 | Quinidine hydrochloride monohydrate | 0.80 | 1.14 |
| **Prestw-281** | 04E02 | Fludrocortisone acetate | 0.81 | 1.11 |
| **Prestw-282** | 04E03 | Fenoterol hydrobromide | 0.82 | 1.15 |

| | | | | |
|---|---|---|---|---|
| **Prestw-283** | 04E04 | Homochlorcyclizine dihydrochloride | 0.72 | 0.81 |
| **Prestw-284** | 04E05 | Diethylcarbamazine citrate | 0.78 | 1.19 |
| **Prestw-285** | 04E06 | Chenodiol | 0.86 | 1.13 |
| **Prestw-286** | 04E07 | Perhexiline maleate | 0.82 | 0.80 |
| **Prestw-287** | 04E08 | Oxybutynin chloride | 0.81 | 1.06 |
| **Prestw-288** | 04E09 | Spiperone | 0.86 | 1.14 |
| **Prestw-289** | 04E10 | Pyrilamine maleate | 0.83 | 1.13 |
| **Prestw-290** | 04E11 | Sulfinpyrazone | 0.77 | 1.09 |
| **Prestw-291** | 04F02 | Dantrolene sodium salt | 0.73 | 0.97 |
| **Prestw-292** | 04F03 | Trazodone hydrochloride | 0.71 | 0.96 |
| **Prestw-293** | 04F04 | Glafenine hydrochloride | 0.79 | 1.09 |
| **Prestw-294** | 04F05 | Pimethixene maleate | 0.73 | 0.81 |
| **Prestw-295** | 04F06 | Pergolide mesylate | 0.85 | 1.26 |
| **Prestw-296** | 04F07 | Acemetacin | 0.77 | 1.05 |
| **Prestw-297** | 04F08 | Benzydamine hydrochloride | 0.83 | 0.99 |
| **Prestw-298** | 04F09 | Fipexide hydrochloride | 0.84 | 1.17 |
| **Prestw-299** | 04F10 | Mifepristone | 0.84 | 1.17 |
| **Prestw-300** | 04F11 | Diperodon hydrochloride | 0.81 | 1.01 |
| **Prestw-301** | 04G02 | Lisinopril | 0.80 | 0.96 |
| **Prestw-302** | 04G03 | Lincomycin hydrochloride | 0.79 | 1.04 |
| **Prestw-303** | 04G04 | Telenzepine dihydrochloride | 0.80 | 1.06 |
| **Prestw-304** | 04G05 | Econazole nitrate | 0.81 | 1.21 |
| **Prestw-305** | 04G06 | Bupivacaine hydrochloride | 0.80 | 1.26 |

| | | | | |
|---|---|---|---|---|
| **Prestw-306** | 04G07 | Clemastine fumarate | 0.77 | 0.86 |
| **Prestw-307** | 04G08 | Oxytetracycline dihydrate | 0.84 | 1.29 |
| **Prestw-308** | 04G09 | Pimozide | 0.85 | 1.16 |
| **Prestw-309** | 04G10 | Amodiaquin dihydrochloride dihydrate | 0.89 | 0.72 |
| **Prestw-310** | 04G11 | Mebeverine hydrochloride | 0.84 | 1.19 |
| **Prestw-311** | 04H02 | Ifenprodil tartrate | 0.75 | 0.98 |
| **Prestw-312** | 04H03 | Flunarizine dihydrochloride | 0.78 | 1.17 |
| **Prestw-313** | 04H04 | Trifluoperazine dihydrochloride | 0.79 | 0.78 |
| **Prestw-314** | 04H05 | Enalapril maleate | 0.85 | 1.05 |
| **Prestw-315** | 04H06 | Minocycline hydrochloride | 0.83 | 1.04 |
| **Prestw-316** | 04H07 | Glibenclamide | 0.81 | 1.19 |
| **Prestw-317** | 04H08 | Guanethidine sulfate | 0.82 | 1.07 |
| **Prestw-318** | 04H09 | Quinacrine dihydrochloride dihydrate | 0.85 | 0.47 |
| **Prestw-319** | 04H10 | Clofilium tosylate | 0.85 | 1.08 |
| **Prestw-320** | 04H11 | Fluphenazine dihydrochloride | 0.85 | 0.76 |
| ***Plate 5*[a]** | | | | |
| **Prestw-321** | 05A02 | Streptomycin sulfate | 1.03 | 1.09 |
| **Prestw-322** | 05A03 | Alfuzosin hydrochloride | 1.00 | 1.14 |
| **Prestw-323** | 05A04 | Chlorpropamide[a] | 1.04 | 1.09 |
| **Prestw-324** | 05A05 | Phenylpropanolamine hydrochloride | 1.03 | 1.08 |
| **Prestw-325** | 05A06 | Ascorbic acid | 1.13 | 1.10 |
| **Prestw-326** | 05A07 | Methyldopa (L,-) | 1.08 | 1.12 |
| **Prestw-327** | 05A08 | Cefoperazone dihydrate | 1.04 | 1.09 |

| | | | | |
|---|---|---|---|---|
| **Prestw-328** | 05A09 | Zoxazolamine | 0.97 | 1.17 |
| **Prestw-329** | 05A10 | Tacrine hydrochloride hydrate | 0.92 | 1.07 |
| **Prestw-330** | 05A11 | Bisoprolol fumarate | 0.97 | 1.27 |
| **Prestw-331** | 05B02 | Tremorine dihydrochloride | 0.87 | 0.79 |
| **Prestw-332** | 05B03 | Practolol | 0.89 | 0.93 |
| **Prestw-333** | 05B04 | Zidovudine, AZT | 0.97 | 0.93 |
| **Prestw-334** | 05B05 | Sulfisoxazole | 0.94 | 0.97 |
| **Prestw-335** | 05B06 | Zaprinast | 1.04 | 1.02 |
| **Prestw-336** | 05B07 | Chlormezanone | 0.92 | 0.91 |
| **Prestw-337** | 05B08 | Procainamide hydrochloride | 0.93 | 0.98 |
| **Prestw-338** | 05B09 | N6-methyladenosine | 0.97 | 1.00 |
| **Prestw-339** | 05B10 | Guanfacine hydrochloride | 1.44 | 1.05 |
| **Prestw-340** | 05B11 | Domperidone | 0.92 | 0.62 |
| **Prestw-341** | 05C02 | Furosemide | 0.93 | 0.93 |
| **Prestw-342** | 05C03 | Methapyrilene hydrochloride | 0.90 | 0.96 |
| **Prestw-343** | 05C04 | Desipramine hydrochloride | 0.78 | 0.65 |
| **Prestw-344** | 05C05 | Clorgyline hydrochloride | 0.88 | 0.98 |
| **Prestw-345** | 05C06 | Clenbuterol hydrochloride | 0.96 | 0.94 |
| **Prestw-346** | 05C07 | Maprotiline hydrochloride | 0.99 | 0.78 |
| **Prestw-347** | 05C08 | Thioguanosine | 0.96 | 1.04 |
| **Prestw-348** | 05C09 | Chlorprothixene hydrochloride | 0.93 | 0.88 |
| **Prestw-349** | 05C10 | Ritodrine hydrochloride | 1.69 | 1.03 |
| **Prestw-350** | 05C11 | Clozapine | 0.96 | 0.73 |

| | | | | |
|---|---|---|---|---|
| **Prestw-351** | 05D02 | Chlorthalidone | 0.91 | 0.88 |
| **Prestw-352** | 05D03 | Dobutamine hydrochloride | 0.86 | 0.91 |
| **Prestw-353** | 05D04 | Moclobemide | 0.95 | 1.00 |
| **Prestw-354** | 05D05 | Clopamide | 0.98 | 0.94 |
| **Prestw-355** | 05D06 | Hycanthone | 0.93 | 0.74 |
| **Prestw-356** | 05D07 | Adenosine 5'-monophosphate monohydrate | 1.03 | 1.12 |
| **Prestw-357** | 05D08 | Amoxicillin | 1.05 | 1.22 |
| **Prestw-358** | 05D09 | Cephalexin monohydrate | 1.07 | 1.17 |
| **Prestw-359** | 05D10 | Dextromethorphan hydrobromide monohydrate | 1.09 | 1.01 |
| **Prestw-360** | 05D11 | Droperidol | 1.04 | 0.95 |
| **Prestw-361** | 05E02 | Bambuterol hydrochloride | 0.97 | 0.88 |
| **Prestw-362** | 05E03 | Betamethasone | 0.95 | 1.09 |
| **Prestw-363** | 05E04 | Colchicine | 1.01 | 0.38 |
| **Prestw-364** | 05E05 | Metergoline | 1.08 | 0.94 |
| **Prestw-365** | 05E06 | Brinzolamide | 1.02 | 0.98 |
| **Prestw-366** | 05E07 | Ambroxol hydrochloride | 0.98 | 1.13 |
| **Prestw-367** | 05E08 | Benfluorex hydrochloride | 0.99 | 1.09 |
| **Prestw-368** | 05E09 | Bepridil hydrochloride | 0.96 | 0.99 |
| **Prestw-369** | 05E10 | Meloxicam | 1.50 | 0.98 |
| **Prestw-370** | 05E11 | Benzbromarone | 1.01 | 0.94 |
| **Prestw-371** | 05F02 | Ketotifen fumarate | 0.88 | 0.78 |
| **Prestw-372** | 05F03 | Debrisoquin sulfate | 0.98 | 0.99 |
| **Prestw-373** | 05F04 | Amethopterin (R,S) | 0.97 | 1.07 |

| | | | | |
|---|---|---|---|---|
| **Prestw-374** | 05F05 | Methylergometrine maleate | 1.09 | 0.99 |
| **Prestw-375** | 05F06 | Methiothepin maleate | 1.14 | 0.63 |
| **Prestw-376** | 05F07 | Clofazimine | 0.98 | 0.98 |
| **Prestw-377** | 05F08 | Nafronyl oxalate | 1.09 | 1.14 |
| **Prestw-378** | 05F09 | Bezafibrate | 1.02 | 1.12 |
| **Prestw-1152** | 05F10 | Nefazodone HCl | 1.34 | 1.04 |
| **Prestw-380** | 05F11 | Clebopride maleate | 1.12 | 0.80 |
| **Prestw-381** | 05G02 | Lidoflazine | 0.80 | 0.53 |
| **Prestw-382** | 05G03 | Betaxolol hydrochloride | 1.00 | 0.96 |
| **Prestw-383** | 05G04 | Nicardipine hydrochloride | 0.36 | 0.45 |
| **Prestw-384** | 05G05 | Probucol | 0.95 | 1.02 |
| **Prestw-385** | 05G06 | Mitoxantrone dihydrochloride | 1.05 | 0.87 |
| **Prestw-386** | 05G07 | GBR 12909 dihydrochloride | 0.98 | 0.96 |
| **Prestw-387** | 05G08 | Carbetapentane citrate | 1.00 | 1.05 |
| **Prestw-388** | 05G09 | Dequalinium dichloride | 1.12 | 0.69 |
| **Prestw-389** | 05G10 | Ketoconazole | 1.00 | 0.85 |
| **Prestw-390** | 05G11 | Fusidic acid sodium salt | 1.04 | 0.76 |
| **Prestw-391** | 05H02 | Terbutaline hemisulfate | 0.91 | 1.01 |
| **Prestw-392** | 05H03 | Ketanserin tartrate hydrate | 0.90 | 1.02 |
| **Prestw-393** | 05H04 | Hemicholinium bromide | 0.84 | 0.99 |
| **Prestw-394** | 05H05 | Kanamycin A sulfate | 0.97 | 1.05 |
| **Prestw-395** | 05H06 | Amikacin hydrate | 0.95 | 0.98 |
| **Prestw-396** | 05H07 | Etoposide | 0.97 | 1.04 |

| | | | | |
|---|---|---|---|---|
| **Prestw-397** | 05H08 | Clomiphene citrate (Z,E) | 0.94 | 0.88 |
| **Prestw-398** | 05H09 | Oxantel pamoate | 0.93 | 1.03 |
| **Prestw-399** | 05H10 | Prochlorperazine dimaleate | 1.03 | 0.81 |
| **Prestw-400** | 05H11 | Hesperidin | 0.95 | 1.22 |
| ***Plate 6*** | | | | |
| **Prestw-401** | 06A02 | Testosterone propionate | 1.02 | 1.14 |
| **Prestw-402** | 06A03 | Arecoline hydrobromide | 1.03 | 1.12 |
| **Prestw-403** | 06A04 | Thyroxine (L) | 1.00 | 0.87 |
| **Prestw-1288** | 06A05 | Idebenone | 1.03 | 0.88 |
| **Prestw-405** | 06A06 | Pepstatin A | 0.99 | 1.01 |
| **Prestw-406** | 06A07 | SR-95639A | 0.99 | 1.06 |
| **Prestw-407** | 06A08 | Adamantamine fumarate | 1.03 | 1.20 |
| **Prestw-408** | 06A09 | Butoconazole nitrate | 1.04 | 1.38 |
| **Prestw-409** | 06A10 | Amiodarone hydrochloride | 1.00 | 1.34 |
| **Prestw-410** | 06A11 | Amphotericin B | 1.04 | 1.50 |
| **Prestw-411** | 06B02 | Androsterone | 0.93 | 1.03 |
| **Prestw-1489** | 06B03 | Amifostine | 0.94 | 1.09 |
| **Prestw-413** | 06B04 | Carbarsone | 0.94 | 0.95 |
| **Prestw-1219** | 06B05 | Amlodipine | 1.07 | 0.72 |
| **Prestw-1147** | 06B06 | Modafinil | 1.00 | 0.99 |
| **Prestw-416** | 06B07 | Bacampicillin hydrochloride | 1.06 | 1.12 |
| **Prestw-1298** | 06B08 | Lamivudine | 0.98 | 1.00 |
| **Prestw-418** | 06B09 | Biotin | 0.99 | 1.27 |

| | | | | |
|---|---|---|---|---|
| **Prestw-419** | 06B10 | Bisacodyl | 1.04 | 1.28 |
| **Prestw-1242** | 06B11 | Erlotinib | 0.99 | 1.25 |
| **Prestw-421** | 06C02 | Suloctidil | 0.99 | 1.03 |
| **Prestw-1368** | 06C03 | Zotepine | 0.97 | 0.82 |
| **Prestw-423** | 06C04 | Carisoprodol | 0.97 | 1.02 |
| **Prestw-424** | 06C05 | Cephalosporanic acid, 7-amino | 0.97 | 1.07 |
| **Prestw-425** | 06C06 | Chicago sky blue 6B | 0.88 | 1.01 |
| **Prestw-426** | 06C07 | Buflomedil hydrochloride | 0.98 | 1.13 |
| **Prestw-1393** | 06C08 | Dibenzepine hydrochloride | 1.06 | 1.09 |
| **Prestw-428** | 06C09 | Roxatidine Acetate HCl | 1.05 | 1.26 |
| **Prestw-429** | 06C10 | Cholecalciferol | 1.00 | 1.29 |
| **Prestw-430** | 06C11 | Cisapride | 0.97 | 1.13 |
| **Prestw-1303** | 06D02 | Pefloxacine | 0.86 | 1.05 |
| **Prestw-432** | 06D03 | Corticosterone | 1.03 | 1.18 |
| **Prestw-433** | 06D04 | Cyanocobalamin | 0.85 | 1.10 |
| **Prestw-434** | 06D05 | Cefadroxil | 0.94 | 1.11 |
| **Prestw-435** | 06D06 | Cyclosporin A | 0.93 | 0.45 |
| **Prestw-436** | 06D07 | Digitoxigenin | 1.12 | 0.78 |
| **Prestw-437** | 06D08 | Digoxin | 1.11 | 0.85 |
| **Prestw-438** | 06D09 | Doxorubicin hydrochloride | 1.01 | 1.06 |
| **Prestw-439** | 06D10 | Carbimazole | 0.95 | 1.22 |
| **Prestw-440** | 06D11 | Epiandrosterone | 1.00 | 1.23 |
| **Prestw-441** | 06E02 | Estradiol-17 beta | 0.88 | 1.04 |

| | | | | |
|---|---|---|---|---|
| **Prestw-1380** | 06E03 | Clobutinol hydrochloride | 0.98 | 1.22 |
| **Prestw-443** | 06E04 | Gabazine | 0.92 | 1.15 |
| **Prestw-1156** | 06E05 | Oxcarbazepine | 0.95 | 1.14 |
| **Prestw-445** | 06E06 | Cyclobenzaprine hydrochloride | 0.96 | 0.87 |
| **Prestw-446** | 06E07 | Carteolol hydrochloride | 0.98 | 1.11 |
| **Prestw-447** | 06E08 | Hydrocortisone base | 1.06 | 1.22 |
| **Prestw-448** | 06E09 | Hydroxytacrine maleate (R,S) | 1.02 | 1.31 |
| **Prestw-449** | 06E10 | Pilocarpine nitrate | 1.02 | 1.33 |
| **Prestw-450** | 06E11 | Dicloxacillin sodium salt | 1.03 | 1.18 |
| **Prestw-451** | 06F02 | Alizapride HCl | 1.00 | 1.20 |
| **Prestw-1161** | 06F03 | Stanozolol | 0.92 | 1.16 |
| **Prestw-1257** | 06F04 | Calcipotriene | 0.96 | 1.31 |
| **Prestw-1429** | 06F05 | Linezolid | 0.98 | 1.11 |
| **Prestw-455** | 06F06 | Mebhydroline 1,5-naphtalenedisulfonate | 0.96 | 1.12 |
| **Prestw-456** | 06F07 | Meclocycline sulfosalicylate | 0.98 | 1.06 |
| **Prestw-457** | 06F08 | Meclozine dihydrochloride | 1.00 | 1.19 |
| **Prestw-458** | 06F09 | Melatonin | 0.98 | 1.25 |
| **Prestw-1251** | 06F10 | Butalbital | 1.08 | 1.43 |
| **Prestw-460** | 06F11 | Dinoprost trometamol | 0.99 | 1.12 |
| **Prestw-461** | 06G02 | Tropisetron HCl | 0.98 | 1.11 |
| **Prestw-462** | 06G03 | Cefixime | 0.93 | 1.13 |
| **Prestw-463** | 06G04 | Metrizamide | 0.92 | 1.27 |
| **Prestw-1323** | 06G05 | Quetiapine | 0.80 | 1.04 |

| | | | | |
|---|---|---|---|---|
| **Prestw-1464** | 06G06 | Tosufloxacin hydrochloride | 0.93 | 1.13 |
| **Prestw-1400** | 06G07 | Efavirenz | 0.81 | 1.01 |
| **Prestw-1157** | 06G08 | Rifapentine | 0.96 | 1.13 |
| **Prestw-468** | 06G09 | Neostigmine bromide | 0.95 | 1.33 |
| **Prestw-469** | 06G10 | Niridazole | 0.92 | 1.19 |
| **Prestw-470** | 06G11 | Ceforanide | 0.90 | 1.12 |
| **Prestw-1358** | 06H02 | Vatalanib | 0.79 | 0.99 |
| **Prestw-1295** | 06H03 | Itopride | 0.89 | 1.03 |
| **Prestw-473** | 06H04 | Cefotetan | 0.93 | 1.01 |
| **Prestw-1254** | 06H05 | Fentiazac | 0.94 | 1.08 |
| **Prestw-475** | 06H06 | Brompheniramine maleate | 0.96 | 0.94 |
| **Prestw-476** | 06H07 | Primaquine diphosphate | 1.07 | 0.95 |
| **Prestw-477** | 06H08 | Progesterone | 0.97 | 1.02 |
| **Prestw-478** | 06H09 | Felodipine | 0.92 | 1.27 |
| **Prestw-1325** | 06H10 | Raclopride | 0.96 | 1.23 |
| **Prestw-1385** | 06H11 | Closantel | 0.97 | 1.41 |
| ***Plate 7*[a]** | | | | |
| **Prestw-481** | 07A02 | Serotonin hydrochloride | 0.78 | 1.33 |
| **Prestw-482** | 07A03 | Cefotiam hydrochloride | 0.90 | 1.05 |
| **Prestw-1336** | 07A04 | Rofecoxib | 0.73 | 1.17 |
| **Prestw-484** | 07A05 | Benperidol | 0.97 | 1.37 |
| **Prestw-485** | 07A06 | Cefaclor | 0.89 | 1.20 |
| **Prestw-486** | 07A07 | Colistin sulfate | 0.80 | 2.02 |

| | | | | |
|---|---|---|---|---|
| **Prestw-487** | 07A08 | Daunorubicin hydrochloride | 0.87 | 1.02 |
| **Prestw-488** | 07A09 | Dosulepin hydrochloride | 0.90 | 1.09 |
| **Prestw-489** | 07A10 | Ceftazidime pentahydrate | 0.88 | 1.44 |
| **Prestw-490** | 07A11 | Iobenguane sulfate | 0.90 | 1.76 |
| **Prestw-491** | 07B02 | Metixene hydrochloride | 0.80 | 0.62 |
| **Prestw-492** | 07B03 | Nitrofural | 0.87 | 1.29 |
| **Prestw-493** | 07B04 | Omeprazole | 0.87 | 1.19 |
| **Prestw-494** | 07B05 | Propylthiouracil | 0.84 | 1.08 |
| **Prestw-495** | 07B06 | Terconazole | 0.95 | 0.63 |
| **Prestw-496** | 07B07 | Tiaprofenic acid | 0.83 | 0.98 |
| **Prestw-497** | 07B08 | Vancomycin hydrochloride | 0.87 | 1.18 |
| **Prestw-498** | 07B09 | Artemisinin | 0.89 | 1.32 |
| **Prestw-499** | 07B10 | Propafenone hydrochloride | 0.88 | 1.51 |
| **Prestw-500** | 07B11 | Ethamivan | 0.79 | 1.81 |
| **Prestw-501** | 07C02 | Vigabatrin | 0.81 | 1.46 |
| **Prestw-502** | 07C03 | Biperiden hydrochloride | 0.75 | 1.42 |
| **Prestw-503** | 07C04 | Cetirizine dihydrochloride | 0.70 | 1.45 |
| **Prestw-504** | 07C05 | Etifenin | 0.91 | 1.18 |
| **Prestw-505** | 07C06 | Metaproterenol sulfate, orciprenaline sulfate | 0.86 | 1.35 |
| **Prestw-506** | 07C07 | Sisomicin sulfate | 0.94 | 1.09 |
| **Prestw-1159** | 07C08 | Sibutramine HCl | 0.97 | 1.09 |
| **Prestw-508** | 07C09 | Resveratrol | 0.80 | 0.91 |
| **Prestw-509** | 07C10 | Bromperidol | 1.03 | 1.10 |

| | | | | |
|---|---|---|---|---|
| **Prestw-510** | 07C11 | Cyclizine hydrochloride | 0.88 | 1.17 |
| **Prestw-511** | 07D02 | Fluoxetine hydrochloride | 0.87 | 1.27 |
| **Prestw-512** | 07D03 | Iohexol | 0.92 | 1.45 |
| **Prestw-513** | 07D04 | Norcyclobenzaprine | 0.89 | 1.02 |
| **Prestw-514** | 07D05 | Pyrazinamide | 0.87 | 1.13 |
| **Prestw-515** | 07D06 | Trimethadione | 0.96 | 1.24 |
| **Prestw-516** | 07D07 | Lovastatin | 0.99 | 1.21 |
| **Prestw-517** | 07D08 | Nystatine | 0.92 | 1.08 |
| **Prestw-518** | 07D09 | Budesonide | 0.83 | 1.56 |
| **Prestw-519** | 07D10 | Imipenem | 0.92 | 1.37 |
| **Prestw-520** | 07D11 | Sulfasalazine | 0.57 | 0.90 |
| **Prestw-1430** | 07E02 | Lofexidine | 0.99 | 1.31 |
| **Prestw-522** | 07E03 | Thiostrepton | 0.84 | 1.40 |
| **Prestw-1169** | 07E04 | Miglitol | 1.06 | 1.61 |
| **Prestw-524** | 07E05 | Tiabendazole | 1.02 | 1.08 |
| **Prestw-525** | 07E06 | Rifampicin | 0.87 | 1.19 |
| **Prestw-526** | 07E07 | Ethionamide | 1.06 | 0.86 |
| **Prestw-527** | 07E08 | Tenoxicam | 0.88 | 1.17 |
| **Prestw-528** | 07E09 | Triflusal | 0.96 | 1.54 |
| **Prestw-529** | 07E10 | Mesoridazine besylate | 0.96 | 1.23 |
| **Prestw-530** | 07E11 | Trolox | 1.05 | 1.32 |
| **Prestw-531** | 07F02 | Pirenperone | 0.99 | 1.54 |
| **Prestw-532** | 07F03 | Isoquinoline, 6,7-dimethoxy-1-methyl-1,2,3,4-tetrahydro, hydrochloride | 1.01 | 1.34 |

| | | | | |
|---|---|---|---|---|
| **Prestw-533** | 07F04 | Phenacetin | 0.87 | 1.45 |
| **Prestw-534** | 07F05 | Atovaquone | 1.05 | 1.53 |
| **Prestw-535** | 07F06 | Methoxamine hydrochloride | 1.02 | 1.29 |
| **Prestw-953** | 07F07 | (S)-(-)-Atenolol | 1.08 | 1.34 |
| **Prestw-537** | 07F08 | Piracetam | 0.81 | 1.41 |
| **Prestw-538** | 07F09 | Phenindione | 1.08 | 1.50 |
| **Prestw-539** | 07F10 | Thiocolchicoside | 1.02 | 1.42 |
| **Prestw-540** | 07F11 | Clorsulon | 0.98 | 1.44 |
| **Prestw-541** | 07G02 | Ciclopirox ethanolamine | 0.91 | 1.23 |
| **Prestw-542** | 07G03 | Probenecid | 0.92 | 1.28 |
| **Prestw-543** | 07G04 | Betahistine mesylate | 0.90 | 1.37 |
| **Prestw-544** | 07G05 | Tobramycin | 1.02 | 1.25 |
| **Prestw-545** | 07G06 | Tetramisole hydrochloride | 0.87 | 1.16 |
| **Prestw-546** | 07G07 | Pregnenolone | 0.89 | 1.06 |
| **Prestw-547** | 07G08 | Molsidomine | 1.00 | 1.32 |
| **Prestw-548** | 07G09 | Chloroquine diphosphate | 1.10 | 1.20 |
| **Prestw-549** | 07G10 | Trimetazidine dihydrochloride | 0.96 | 1.49 |
| **Prestw-550** | 07G11 | Parthenolide | 1.03 | 0.93 |
| **Prestw-551** | 07H02 | Hexetidine | 0.83 | 1.27 |
| **Prestw-552** | 07H03 | Selegiline hydrochloride | 0.93 | 1.22 |
| **Prestw-553** | 07H04 | Pentamidine isethionate | 1.00 | 1.28 |
| **Prestw-554** | 07H05 | Tolazamide | 0.93 | 1.19 |
| **Prestw-555** | 07H06 | Nifuroxazide | 0.86 | 1.11 |

| | | | | |
|---|---|---|---|---|
| **Prestw-1144** | 07H07 | Mirtazapine | 0.93 | 1.00 |
| **Prestw-557** | 07H08 | Dirithromycin | 0.88 | 1.03 |
| **Prestw-558** | 07H09 | Gliclazide | 0.88 | 1.30 |
| **Prestw-559** | 07H10 | DO 897/99 | 0.96 | 1.28 |
| **Prestw-560** | 07H11 | Prenylamine lactate | 0.95 | 1.13 |
| ***Plate 8*** | | | | |
| **Prestw-1188** | 08A02 | Ziprasidone Hydrochloride | 1.04 | 0.98 |
| **Prestw-1441** | 08A03 | Mevastatin | 1.04 | 0.92 |
| **Prestw-1322** | 08A04 | Pyridostigmine iodid | 1.03 | 1.06 |
| **Prestw-1491** | 08A05 | Pentobarbital | 1.03 | 1.23 |
| **Prestw-565** | 08A06 | Atropine sulfate monohydrate | 0.95 | 1.32 |
| **Prestw-566** | 08A07 | Eserine sulfate, physostigmine sulfate | 1.03 | 1.02 |
| **Prestw-1139** | 08A08 | Itraconazole | 0.93 | 0.99 |
| **Prestw-1174** | 08A09 | Acarbose | 0.98 | 1.48 |
| **Prestw-1403** | 08A10 | Entacapone | 1.03 | 1.44 |
| **Prestw-1449** | 08A11 | Nicotinamide | 0.97 | 1.85 |
| **Prestw-571** | 08B02 | Tetracaïne hydrochloride | 0.99 | 1.22 |
| **Prestw-572** | 08B03 | Mometasone furoate | 1.07 | 1.17 |
| **Prestw-1467** | 08B04 | Troglitazone | 1.02 | 1.13 |
| **Prestw-574** | 08B05 | Dacarbazine | 1.01 | 1.12 |
| **Prestw-1351** | 08B06 | Tenatoprazole | 0.99 | 1.15 |
| **Prestw-576** | 08B07 | Acetopromazine maleate salt | 1.03 | 0.84 |
| **Prestw-1271** | 08B08 | Escitalopram | 0.91 | 0.89 |

| | | | | |
|---|---|---|---|---|
| **Prestw-1158** | 08B09 | Ropinirole HCl | 0.95 | 1.28 |
| **Prestw-1297** | 08B10 | Lacidipine | 1.04 | 1.22 |
| **Prestw-1228** | 08B11 | Argatroban | 0.93 | 1.46 |
| **Prestw-1328** | 08C02 | Reboxetine mesylate | 0.98 | 1.07 |
| **Prestw-582** | 08C03 | Lobelanidine hydrochloride | 0.95 | 1.26 |
| **Prestw-583** | 08C04 | Papaverine hydrochloride | 0.83 | 1.11 |
| **Prestw-584** | 08C05 | Yohimbine hydrochloride | 1.02 | 1.14 |
| **Prestw-585** | 08C06 | Lobeline alpha (-) hydrochoride | 0.93 | 1.02 |
| **Prestw-1211** | 08C07 | Alfacalcidol | 1.00 | 1.09 |
| **Prestw-587** | 08C08 | Cilostazol | 0.97 | 0.99 |
| **Prestw-588** | 08C09 | Galanthamine hydrobromide | 0.98 | 1.13 |
| **Prestw-1130** | 08C10 | Azelastine HCl | 0.95 | 1.04 |
| **Prestw-1409** | 08C11 | Etretinate | 1.01 | 1.34 |
| **Prestw-1274** | 08D02 | Emedastine | 1.09 | 1.28 |
| **Prestw-1407** | 08D03 | Etofenamate | 0.81 | 1.28 |
| **Prestw-1369** | 08D04 | Zaleplon | 0.96 | 1.24 |
| **Prestw-594** | 08D05 | Diclofenac sodium | 1.04 | 1.17 |
| **Prestw-1410** | 08D06 | Exemestane | 0.93 | 1.09 |
| **Prestw-596** | 08D07 | Convolamine hydrochloride | 0.99 | 0.97 |
| **Prestw-1183** | 08D08 | Temozolomide | 0.97 | 1.10 |
| **Prestw-598** | 08D09 | Xylazine | 1.02 | 1.22 |
| **Prestw-1132** | 08D10 | Celiprolol HCl | 1.00 | 1.11 |
| **Prestw-1367** | 08D11 | Zopiclone | 0.97 | 1.18 |

| | | | | |
|---|---|---|---|---|
| **Prestw-1198** | 08E02 | Tranilast | 0.91 | 1.16 |
| **Prestw-1182** | 08E03 | Tizanidine HCl | 1.03 | 1.42 |
| **Prestw-1364** | 08E04 | Zafirlukast | 0.94 | 1.54 |
| **Prestw-1252** | 08E05 | Butenafine | 0.93 | 1.14 |
| **Prestw-1121** | 08E06 | Carbadox | 0.99 | 1.09 |
| **Prestw-1331** | 08E07 | Rimantadine | 0.99 | 0.98 |
| **Prestw-607** | 08E08 | Eburnamonine (-) | 0.96 | 1.22 |
| **Prestw-1460** | 08E09 | Oxibendazol | 1.04 | 1.23 |
| **Prestw-1292** | 08E10 | Ipsapirone | 0.96 | 1.29 |
| **Prestw-610** | 08E11 | Harmaline hydrochloride dihydrate | 0.97 | 1.11 |
| **Prestw-611** | 08F02 | Harmalol hydrochloride dihydrate | 0.94 | 1.23 |
| **Prestw-612** | 08F03 | Harmol hydrochloride monohydrate | 1.01 | 1.30 |
| **Prestw-613** | 08F04 | Harmine hydrochloride | 0.88 | 1.10 |
| **Prestw-1177** | 08F05 | Carbidopa | 0.93 | 1.08 |
| **Prestw-615** | 08F06 | Chrysene-1,4-quinone | 1.00 | 1.06 |
| **Prestw-616** | 08F07 | Demecarium bromide | 0.99 | 1.07 |
| **Prestw-617** | 08F08 | Quipazine dimaleate salt | 1.01 | 1.14 |
| **Prestw-1127** | 08F09 | Acipimox | 0.98 | 1.17 |
| **Prestw-619** | 08F10 | Diflorasone Diacetate | 1.01 | 1.40 |
| **Prestw-620** | 08F11 | Harmane hydrochloride | 0.98 | 1.22 |
| **Prestw-621** | 08G02 | Methoxy-6-harmalan | 0.85 | 1.05 |
| **Prestw-1217** | 08G03 | Amisulpride | 0.94 | 1.19 |
| **Prestw-623** | 08G04 | Pyridoxine hydrochloride | 0.93 | 1.23 |

| | | | | |
|---|---|---|---|---|
| **Prestw-1469** | 08G05 | Mercaptopurine | 0.91 | 1.03 |
| **Prestw-1134** | 08G06 | Cytarabine | 0.91 | 0.90 |
| **Prestw-626** | 08G07 | Racecadotril | 0.89 | 1.08 |
| **Prestw-627** | 08G08 | Folic acid | 0.97 | 1.01 |
| **Prestw-1129** | 08G09 | Benazepril HCl | 0.92 | 1.40 |
| **Prestw-1178** | 08G10 | Aniracetam | 0.92 | 1.27 |
| **Prestw-630** | 08G11 | Dimethisoquin hydrochloride | 0.92 | 1.45 |
| **Prestw-1210** | 08H02 | Alendronate sodium | 0.91 | 1.03 |
| **Prestw-632** | 08H03 | Dipivefrin hydrochloride | 0.88 | 1.00 |
| **Prestw-633** | 08H04 | Thiorphan | 0.88 | 1.07 |
| **Prestw-1463** | 08H05 | Tomoxetine hydrochloride | 0.83 | 0.83 |
| **Prestw-1299** | 08H06 | Lapatinib ditosylate | 0.94 | 1.08 |
| **Prestw-1488** | 08H07 | Penciclovir | 0.87 | 1.12 |
| **Prestw-1427** | 08H08 | Levetiracetam | 0.94 | 1.02 |
| **Prestw-1392** | 08H09 | Dexfenfluramine hydrochloride | 0.88 | 1.27 |
| **Prestw-1408** | 08H10 | Etoricoxib | 0.90 | 1.19 |
| **Prestw-1341** | 08H11 | Sertindole | 0.86 | 0.67 |
| ***Plate 9*** | | | | |
| **Prestw-641** | 09A02 | Sulmazole | 0.86 | 1.07 |
| **Prestw-1270** | 09A03 | Gefitinib | 0.87 | 0.96 |
| **Prestw-643** | 09A04 | Flunisolide | 0.93 | 0.99 |
| **Prestw-644** | 09A05 | N-Acetyl-DL-homocysteine Thiolactone | 0.90 | 1.00 |
| **Prestw-645** | 09A06 | Flurandrenolide | 0.89 | 1.14 |

| | | | | |
|---|---|---|---|---|
| **Prestw-1125** | 09A07 | Oxiconazole Nitrate | 0.89 | 0.87 |
| **Prestw-1166** | 09A08 | Rebamipide | 0.89 | 1.02 |
| **Prestw-1154** | 09A09 | Nilvadipine | 0.81 | 1.21 |
| **Prestw-649** | 09A10 | Etanidazole | 0.81 | 1.24 |
| **Prestw-650** | 09A11 | Butirosin disulfate salt | 0.88 | 1.60 |
| **Prestw-651** | 09B02 | Glimepiride | 0.82 | 0.94 |
| **Prestw-652** | 09B03 | Picrotoxinin | 0.92 | 0.99 |
| **Prestw-653** | 09B04 | Mepenzolate bromide | 0.90 | 1.09 |
| **Prestw-654** | 09B05 | Benfotiamine | 0.87 | 1.00 |
| **Prestw-655** | 09B06 | Halcinonide | 0.87 | 1.05 |
| **Prestw-656** | 09B07 | Lanatoside C | 0.92 | 0.71 |
| **Prestw-657** | 09B08 | Benzamil hydrochloride | 0.93 | 0.78 |
| **Prestw-658** | 09B09 | Suxibuzone | 0.84 | 1.20 |
| **Prestw-659** | 09B10 | 6-Furfurylaminopurine | 0.87 | 1.10 |
| **Prestw-660** | 09B11 | Avermectin B1a | 0.85 | 1.16 |
| **Prestw-1317** | 09C02 | Pranlukast | 0.88 | 0.97 |
| **Prestw-1477** | 09C03 | Penicillamine | 0.91 | 1.12 |
| **Prestw-1365** | 09C04 | Zileuton | 0.91 | 0.98 |
| **Prestw-1432** | 09C05 | Loratadine | 0.85 | 0.96 |
| **Prestw-1201** | 09C06 | Clindamycin Phosphate | 0.89 | 1.06 |
| **Prestw-666** | 09C07 | Nisoldipine | 0.84 | 0.84 |
| **Prestw-667** | 09C08 | Foliosidine | 0.92 | 0.83 |
| **Prestw-1165** | 09C09 | Acitretin | 0.87 | 1.08 |

| | | | | |
|---|---|---|---|---|
| **Prestw-1162** | 09C10 | Zonisamide | 0.95 | 1.27 |
| **Prestw-1173** | 09C11 | Irsogladine Maleate | 0.90 | 1.06 |
| **Prestw-671** | 09D02 | Dydrogesterone | 0.93 | 1.19 |
| **Prestw-1346** | 09D03 | Sumatriptan succinate | 0.88 | 1.21 |
| **Prestw-1456** | 09D04 | Opipramol dihydrochloride | 0.82 | 0.86 |
| **Prestw-1447** | 09D05 | Nalidixic acid sodium salt | 0.94 | 1.05 |
| **Prestw-1475** | 09D06 | Oxacillin Na | 0.96 | 1.05 |
| **Prestw-676** | 09D07 | Beta-Escin | 0.91 | 0.97 |
| **Prestw-1496** | 09D08 | Tiludronate disodium | 0.91 | 0.90 |
| **Prestw-1349** | 09D09 | Tazobactam | 0.87 | 1.15 |
| **Prestw-1285** | 09D10 | Ibandronate | 0.93 | 1.02 |
| **Prestw-1363** | 09D11 | Warfarin | 0.98 | 1.16 |
| **Prestw-1318** | 09E02 | Pranoprofen | 0.91 | 1.14 |
| **Prestw-1340** | 09E03 | Secnidazole | 0.93 | 1.21 |
| **Prestw-683** | 09E04 | Pempidine tartrate | 0.97 | 1.33 |
| **Prestw-1381** | 09E05 | Clodronate | 0.92 | 1.14 |
| **Prestw-685** | 09E06 | Nitrarine dihydrochloride | 0.98 | 0.83 |
| **Prestw-1194** | 09E07 | Thimerosal | 0.95 | 0.25 |
| **Prestw-1465** | 09E08 | Tramadol hydrochloride | 0.96 | 0.97 |
| **Prestw-688** | 09E09 | Estropipate | 0.96 | 1.04 |
| **Prestw-1253** | 09E10 | Butylscopolammonium (n-) bromide | 0.93 | 1.08 |
| **Prestw-1494** | 09E11 | Irinotecan Hydrochloride | 0.94 | 1.02 |
| **Prestw-1353** | 09F02 | Tylosin | 1.00 | 1.09 |

| | | | | |
|---|---|---|---|---|
| **Prestw-692** | 09F03 | Citalopram Hydrobromide | 0.96 | 1.12 |
| **Prestw-693** | 09F04 | Promazine hydrochloride | 0.99 | 0.83 |
| **Prestw-694** | 09F05 | Sulfamerazine | 0.97 | 1.05 |
| **Prestw-1170** | 09F06 | Venlafaxine | 1.03 | 0.99 |
| **Prestw-696** | 09F07 | Ethotoin | 1.01 | 1.03 |
| **Prestw-697** | 09F08 | 3-alpha-Hydroxy-5-beta-androstan-17-one | 0.91 | 1.15 |
| **Prestw-698** | 09F09 | Tetrahydrozoline hydrochloride | 0.96 | 1.21 |
| **Prestw-699** | 09F10 | Hexestrol | 1.00 | 1.32 |
| **Prestw-700** | 09F11 | Cefmetazole sodium salt | 0.96 | 1.07 |
| **Prestw-701** | 09G02 | Trihexyphenidyl-D,L Hydrochloride | 0.91 | 1.06 |
| **Prestw-702** | 09G03 | Succinylsulfathiazole | 0.88 | 0.98 |
| **Prestw-703** | 09G04 | Famprofazone | 0.86 | 1.00 |
| **Prestw-704** | 09G05 | Bromopride | 1.03 | 0.99 |
| **Prestw-705** | 09G06 | Methyl benzethonium chloride | 1.32 | 1.07 |
| **Prestw-706** | 09G07 | Chlorcyclizine hydrochloride | 0.77 | 0.84 |
| **Prestw-707** | 09G08 | Diphenylpyraline hydrochloride | 0.88 | 0.90 |
| **Prestw-708** | 09G09 | Benzethonium chloride | 1.09 | 1.14 |
| **Prestw-709** | 09G10 | Trioxsalen | 0.96 | 1.14 |
| **Prestw-1136** | 09G11 | Doxofylline | 0.93 | 1.18 |
| **Prestw-711** | 09H02 | Sulfabenzamide | 0.88 | 0.91 |
| **Prestw-712** | 09H03 | Benzocaine | 0.87 | 0.94 |
| **Prestw-713** | 09H04 | Dipyrone | 0.92 | 1.00 |
| **Prestw-714** | 09H05 | Isosorbide dinitrate | 0.90 | 0.81 |

| | | | | |
|---|---|---|---|---|
| **Prestw-715** | 09H06 | Sulfachloropyridazine | 0.85 | 0.88 |
| **Prestw-716** | 09H07 | Pramoxine hydrochloride | 0.94 | 1.01 |
| **Prestw-717** | 09H08 | Finasteride | 0.88 | 0.97 |
| **Prestw-718** | 09H09 | Fluorometholone | 0.88 | 1.08 |
| **Prestw-719** | 09H10 | Cephalothin sodium salt | 0.88 | 1.08 |
| **Prestw-720** | 09H11 | Cefuroxime sodium salt | 0.90 | 1.11 |
| ***Plate 10*** | | | | |
| **Prestw-721** | 10A02 | Althiazide | 1.01 | 0.97 |
| **Prestw-722** | 10A03 | Isopyrin hydrochloride | 1.02 | 0.99 |
| **Prestw-723** | 10A04 | Phenethicillin potassium salt | 1.01 | 0.97 |
| **Prestw-724** | 10A05 | Sulfamethoxypyridazine | 0.95 | 0.87 |
| **Prestw-725** | 10A06 | Deferoxamine mesylate | 0.96 | 0.91 |
| **Prestw-726** | 10A07 | Mephentermine hemisulfate | 0.83 | 0.96 |
| **Prestw-1140** | 10A08 | Liranaftate | 0.93 | 0.99 |
| **Prestw-728** | 10A09 | Sulfadimethoxine | 0.89 | 0.88 |
| **Prestw-729** | 10A10 | Sulfanilamide | 0.80 | 0.94 |
| **Prestw-730** | 10A11 | Balsalazide Sodium | 0.84 | 0.95 |
| **Prestw-731** | 10B02 | Sulfaquinoxaline sodium salt | 0.94 | 1.00 |
| **Prestw-732** | 10B03 | Streptozotocin | 0.99 | 1.09 |
| **Prestw-733** | 10B04 | Metoprolol-(+,-) (+)-tartrate salt | 1.01 | 1.04 |
| **Prestw-734** | 10B05 | Flumethasone | 0.92 | 1.08 |
| **Prestw-735** | 10B06 | Flecainide acetate | 0.99 | 1.02 |
| **Prestw-736** | 10B07 | Cefazolin sodium salt | 0.92 | 1.04 |

| | | | | |
|---|---|---|---|---|
| **Prestw-737** | 10B08 | Atractyloside potassium salt | 0.90 | 1.11 |
| **Prestw-738** | 10B09 | Folinic acid calcium salt | 0.92 | 0.99 |
| **Prestw-739** | 10B10 | Levonordefrin | 0.90 | 1.02 |
| **Prestw-740** | 10B11 | Ebselen | 0.91 | 1.15 |
| **Prestw-741** | 10C02 | Nadide | 0.89 | 1.11 |
| **Prestw-742** | 10C03 | Sulfamethizole | 0.93 | 1.09 |
| **Prestw-743** | 10C04 | Medrysone | 0.93 | 1.03 |
| **Prestw-744** | 10C05 | Flunixin meglumine | 0.92 | 1.02 |
| **Prestw-745** | 10C06 | Spiramycin | 0.98 | 1.08 |
| **Prestw-746** | 10C07 | Glycopyrrolate | 1.01 | 1.09 |
| **Prestw-747** | 10C08 | Cefamandole sodium salt | 0.90 | 1.02 |
| **Prestw-748** | 10C09 | Monensin sodium salt | 0.97 | 1.19 |
| **Prestw-749** | 10C10 | Isoetharine mesylate salt | 0.85 | 1.03 |
| **Prestw-750** | 10C11 | Mevalonic-D, L acid lactone | 0.91 | 1.08 |
| **Prestw-751** | 10D02 | Terazosin hydrochloride | 0.87 | 1.10 |
| **Prestw-752** | 10D03 | Phenazopyridine hydrochloride | 0.86 | 1.05 |
| **Prestw-753** | 10D04 | Demeclocycline hydrochloride | 0.90 | 1.00 |
| **Prestw-754** | 10D05 | Fenoprofen calcium salt dihydrate | 0.93 | 1.05 |
| **Prestw-755** | 10D06 | Piperacillin sodium salt | 0.95 | 1.11 |
| **Prestw-756** | 10D07 | Diethylstilbestrol | 0.90 | 1.01 |
| **Prestw-757** | 10D08 | Chlorotrianisene | 0.90 | 1.04 |
| **Prestw-758** | 10D09 | Ribostamycin sulfate salt | 0.88 | 0.95 |
| **Prestw-759** | 10D10 | Methacholine chloride | 0.93 | 0.94 |

| | | | | |
|---|---|---|---|---|
| **Prestw-760** | 10D11 | Pipenzolate bromide | 0.81 | 0.96 |
| **Prestw-761** | 10E02 | Butamben | 0.87 | 1.08 |
| **Prestw-762** | 10E03 | Sulfapyridine | 0.89 | 0.98 |
| **Prestw-763** | 10E04 | Meclofenoxate hydrochloride | 0.92 | 1.14 |
| **Prestw-764** | 10E05 | Furaltadone hydrochloride | 0.92 | 0.96 |
| **Prestw-765** | 10E06 | Ethoxyquin | 0.83 | 0.92 |
| **Prestw-766** | 10E07 | Tinidazole | 0.91 | 1.06 |
| **Prestw-767** | 10E08 | Guanadrel sulfate | 0.88 | 0.96 |
| **Prestw-768** | 10E09 | Vidarabine | 0.91 | 1.01 |
| **Prestw-769** | 10E10 | Sulfameter | 0.86 | 0.88 |
| **Prestw-770** | 10E11 | Isopropamide iodide | 0.88 | 0.96 |
| **Prestw-771** | 10F02 | Alclometasone dipropionate | 0.85 | 1.15 |
| **Prestw-772** | 10F03 | Leflunomide | 0.85 | 0.94 |
| **Prestw-773** | 10F04 | Norgestrel-(-)-D | 0.85 | 0.96 |
| **Prestw-774** | 10F05 | Fluocinonide | 0.91 | 1.14 |
| **Prestw-775** | 10F06 | Sulfamethazine sodium salt | 0.88 | 1.06 |
| **Prestw-776** | 10F07 | Guaifenesin | 0.96 | 1.04 |
| **Prestw-777** | 10F08 | Alexidine dihydrochloride | 16.01 | 6.74 |
| **Prestw-778** | 10F09 | Proadifen hydrochloride | 0.81 | 0.95 |
| **Prestw-779** | 10F10 | Zomepirac sodium salt | 0.81 | 0.95 |
| **Prestw-780** | 10F11 | Cinoxacin | 0.79 | 1.02 |
| **Prestw-781** | 10G02 | Clobetasol propionate | 0.85 | 1.02 |
| **Prestw-782** | 10G03 | Podophyllotoxin | 0.94 | 0.68 |

| | | | | |
|---|---|---|---|---|
| **Prestw-783** | 10G04 | Clofibric acid | 0.84 | 1.03 |
| **Prestw-784** | 10G05 | Bendroflumethiazide | 0.88 | 1.04 |
| **Prestw-785** | 10G06 | Dicumarol | 0.91 | 1.11 |
| **Prestw-786** | 10G07 | Methimazole | 0.84 | 0.93 |
| **Prestw-787** | 10G08 | Merbromin | 0.74 | 0.89 |
| **Prestw-788** | 10G09 | Hexylcaine hydrochloride | 0.81 | 1.00 |
| **Prestw-789** | 10G10 | Drofenine hydrochloride | 0.78 | 0.97 |
| **Prestw-790** | 10G11 | Cycloheximide | 0.80 | 0.99 |
| **Prestw-791** | 10H02 | (R) -Naproxen sodium salt | 0.77 | 0.93 |
| **Prestw-792** | 10H03 | Propidium iodide | 0.74 | 0.85 |
| **Prestw-793** | 10H04 | Cloperastine hydrochloride | 0.82 | 0.80 |
| **Prestw-794** | 10H05 | Eucatropine hydrochloride | 0.80 | 0.82 |
| **Prestw-795** | 10H06 | Isocarboxazid | 0.83 | 0.79 |
| **Prestw-796** | 10H07 | Lithocholic acid | 0.78 | 0.78 |
| **Prestw-797** | 10H08 | Methotrimeprazine maleat salt | 0.84 | 0.81 |
| **Prestw-798** | 10H09 | Dienestrol | 0.84 | 0.86 |
| **Prestw-799** | 10H10 | Pridinol methanesulfonate salt | 0.78 | 0.95 |
| **Prestw-800** | 10H11 | Amrinone | 0.77 | 0.96 |
| ***Plate 11*** | | | | |
| **Prestw-801** | 11A02 | Carbinoxamine maleate salt | 0.91 | 1.04 |
| **Prestw-802** | 11A03 | Methazolamide | 0.99 | 1.00 |
| **Prestw-803** | 11A04 | Pyrithyldione | 1.01 | 1.12 |
| **Prestw-804** | 11A05 | Spectinomycin dihydrochloride | 0.99 | 0.99 |

| | | | | |
|---|---|---|---|---|
| **Prestw-805** | 11A06 | Piromidic acid | 1.01 | 1.09 |
| **Prestw-806** | 11A07 | Trimipramine maleate salt | 0.91 | 0.95 |
| **Prestw-807** | 11A08 | Chloropyramine hydrochloride | 0.95 | 1.04 |
| **Prestw-808** | 11A09 | Furazolidone | 0.97 | 1.12 |
| **Prestw-809** | 11A10 | Dichlorphenamide | 0.92 | 1.26 |
| **Prestw-810** | 11A11 | Sulconazole nitrate | 1.02 | 1.18 |
| **Prestw-1233** | 11B02 | Auranofin | 1.02 | 0.49 |
| **Prestw-812** | 11B03 | Cromolyn disodium salt | 0.98 | 0.87 |
| **Prestw-813** | 11B04 | Bucladesine sodium salt | 0.94 | 0.88 |
| **Prestw-814** | 11B05 | Cefsulodin sodium salt | 0.96 | 0.84 |
| **Prestw-815** | 11B06 | Fosfosal | 0.96 | 1.10 |
| **Prestw-816** | 11B07 | Suprofen | 0.92 | 1.03 |
| **Prestw-817** | 11B08 | Catechin-(+,-) hydrate | 0.92 | 1.08 |
| **Prestw-818** | 11B09 | Nadolol | 0.94 | 1.19 |
| **Prestw-819** | 11B10 | Moxalactam disodium salt | 1.03 | 1.16 |
| **Prestw-820** | 11B11 | Aminophylline | 0.97 | 0.99 |
| **Prestw-821** | 11C02 | Azlocillin sodium salt | 0.98 | 1.07 |
| **Prestw-822** | 11C03 | Clidinium bromide | 0.94 | 0.96 |
| **Prestw-823** | 11C04 | Sulfamonomethoxine | 0.99 | 0.99 |
| **Prestw-824** | 11C05 | Benzthiazide | 1.01 | 1.03 |
| **Prestw-825** | 11C06 | Trichlormethiazide | 0.97 | 1.23 |
| **Prestw-826** | 11C07 | Oxalamine citrate salt | 0.92 | 1.18 |
| **Prestw-827** | 11C08 | Propantheline bromide | 0.94 | 1.19 |

| | | | | |
|---|---|---|---|---|
| **Prestw-1361** | 11C09 | Viloxazine hydrochloride | 0.98 | 0.99 |
| **Prestw-829** | 11C10 | Dimethadione | 0.92 | 1.01 |
| **Prestw-830** | 11C11 | Ethaverine hydrochloride | 0.89 | 0.93 |
| **Prestw-831** | 11D02 | Butacaine | 0.93 | 0.97 |
| **Prestw-832** | 11D03 | Cefoxitin sodium salt | 0.96 | 0.99 |
| **Prestw-833** | 11D04 | Ifosfamide | 0.95 | 1.11 |
| **Prestw-834** | 11D05 | Novobiocin sodium salt | 0.85 | 1.09 |
| **Prestw-835** | 11D06 | Tetrahydroxy-1,4-quinone monohydrate | 1.03 | 1.19 |
| **Prestw-836** | 11D07 | Indoprofen | 0.83 | 1.26 |
| **Prestw-837** | 11D08 | Carbenoxolone disodium salt | 0.90 | 1.01 |
| **Prestw-838** | 11D09 | Iocetamic acid | 0.93 | 1.13 |
| **Prestw-839** | 11D10 | Ganciclovir | 0.89 | 1.20 |
| **Prestw-840** | 11D11 | Ethopropazine hydrochloride | 0.94 | 0.78 |
| **Prestw-1455** | 11E02 | Olanzapine | 0.95 | 0.88 |
| **Prestw-842** | 11E03 | Trimeprazine tartrate | 0.85 | 0.63 |
| **Prestw-843** | 11E04 | Nafcillin sodium salt monohydrate | 0.85 | 1.07 |
| **Prestw-844** | 11E05 | Procyclidine hydrochloride | 0.86 | 0.95 |
| **Prestw-845** | 11E06 | Amiprilose hydrochloride | 0.92 | 1.10 |
| **Prestw-846** | 11E07 | Ethynylestradiol 3-methyl ether | 0.97 | 1.05 |
| **Prestw-847** | 11E08 | (-) -Levobunolol hydrochloride | 0.91 | 1.06 |
| **Prestw-848** | 11E09 | Iodixanol | 0.90 | 1.10 |
| **Prestw-849** | 11E10 | Rolitetracycline | 0.78 | 0.97 |
| **Prestw-850** | 11E11 | Equilin | 0.90 | 0.87 |

| | | | | |
|---|---|---|---|---|
| **Prestw-851** | 11F02 | Paroxetine Hydrochloride | 0.95 | 0.78 |
| **Prestw-1454** | 11F03 | Nylidrin | 0.89 | 0.91 |
| **Prestw-853** | 11F04 | Liothyronine | 0.87 | 1.07 |
| **Prestw-854** | 11F05 | Roxithromycin | 0.91 | 1.01 |
| **Prestw-855** | 11F06 | Beclomethasone dipropionate | 0.89 | 1.16 |
| **Prestw-856** | 11F07 | Tolmetin sodium salt dihydrate | 0.97 | 1.11 |
| **Prestw-857** | 11F08 | (+) -Levobunolol hydrochloride | 0.97 | 1.10 |
| **Prestw-858** | 11F09 | Doxazosin mesylate | 0.87 | 0.92 |
| **Prestw-859** | 11F10 | Fluvastatin sodium salt | 0.88 | 0.98 |
| **Prestw-860** | 11F11 | Methylhydantoin-5-(L) | 0.91 | 0.87 |
| **Prestw-861** | 11G02 | Gabapentin | 0.84 | 0.94 |
| **Prestw-862** | 11G03 | Raloxifene hydrochloride | 0.89 | 0.82 |
| **Prestw-863** | 11G04 | Etidronic acid, disodium salt | 0.92 | 0.91 |
| **Prestw-864** | 11G05 | Methylhydantoin-5-(D) | 0.88 | 1.04 |
| **Prestw-865** | 11G06 | Simvastatin | 0.88 | 0.94 |
| **Prestw-866** | 11G07 | Azacytidine-5 | 0.86 | 0.91 |
| **Prestw-867** | 11G08 | Paromomycin sulfate | 0.83 | 1.10 |
| **Prestw-868** | 11G09 | Acetaminophen | 0.89 | 1.04 |
| **Prestw-869** | 11G10 | Phthalylsulfathiazole | 0.87 | 0.97 |
| **Prestw-870** | 11G11 | Luteolin | 0.81 | 0.96 |
| **Prestw-871** | 11H02 | Iopamidol | 0.88 | 1.11 |
| **Prestw-872** | 11H03 | Iopromide | 0.82 | 1.10 |
| **Prestw-873** | 11H04 | Theophylline monohydrate | 0.86 | 0.94 |

| | | | | |
|---|---|---|---|---|
| **Prestw-874** | 11H05 | Theobromine | 0.90 | 0.97 |
| **Prestw-875** | 11H06 | Reserpine | 0.81 | 0.76 |
| **Prestw-1239** | 11H07 | Bicalutamide | 0.84 | 0.90 |
| **Prestw-877** | 11H08 | Scopolamine hydrochloride | 0.88 | 0.89 |
| **Prestw-878** | 11H09 | Ioversol | 0.84 | 0.96 |
| **Prestw-1495** | 11H10 | Rabeprazole | 0.79 | 0.93 |
| **Prestw-880** | 11H11 | Carbachol | 0.91 | 0.97 |
| ***Plate 12*** | | | | |
| **Prestw-881** | 12A02 | Niacin | 1.03 | 1.17 |
| **Prestw-882** | 12A03 | Bemegride | 1.04 | 1.14 |
| **Prestw-883** | 12A04 | Digoxigenin | 1.03 | 0.86 |
| **Prestw-884** | 12A05 | Meglumine | 0.98 | 1.11 |
| **Prestw-885** | 12A06 | Cantharidin | 0.98 | 1.03 |
| **Prestw-886** | 12A07 | Clioquinol | 0.93 | 1.18 |
| **Prestw-887** | 12A08 | Oxybenzone | 0.94 | 1.14 |
| **Prestw-888** | 12A09 | Promethazine hydrochloride | 0.89 | 1.03 |
| **Prestw-1167** | 12A10 | Diacerein | 0.94 | 1.06 |
| **Prestw-1137** | 12A11 | Esmolol hydrochloride | 0.90 | 1.13 |
| **Prestw-1486** | 12B02 | Cortisol acetate | 0.97 | 0.93 |
| **Prestw-1416** | 12B03 | Flubendazol | 0.97 | 0.95 |
| **Prestw-893** | 12B04 | Felbinac | 1.00 | 0.88 |
| **Prestw-894** | 12B05 | Butylparaben | 1.03 | 0.86 |
| **Prestw-895** | 12B06 | Aminohippuric acid | 0.91 | 0.90 |

| | | | | |
|---|---|---|---|---|
| **Prestw-896** | 12B07 | N-Acetyl-L-leucine | 1.02 | 0.92 |
| **Prestw-897** | 12B08 | Pipemidic acid | 0.98 | 0.95 |
| **Prestw-898** | 12B09 | Dioxybenzone | 0.95 | 0.79 |
| **Prestw-899** | 12B10 | Adrenosterone | 0.99 | 0.75 |
| **Prestw-900** | 12B11 | Methylatropine nitrate | 1.00 | 0.98 |
| **Prestw-901** | 12C02 | Hymecromone | 0.96 | 0.97 |
| **Prestw-902** | 12C03 | Caffeic acid | 0.90 | 0.94 |
| **Prestw-903** | 12C04 | Diloxanide furoate | 0.95 | 0.94 |
| **Prestw-904** | 12C05 | Metyrapone | 0.93 | 0.91 |
| **Prestw-905** | 12C06 | Urapidil hydrochloride | 0.96 | 0.89 |
| **Prestw-906** | 12C07 | Fluspirilen | 0.95 | 0.78 |
| **Prestw-907** | 12C08 | S-(+)-ibuprofen | 1.00 | 0.86 |
| **Prestw-908** | 12C09 | Ethynodiol diacetate | 0.99 | 0.82 |
| **Prestw-909** | 12C10 | Nabumetone | 0.96 | 0.74 |
| **Prestw-910** | 12C11 | Nisoxetine hydrochloride | 0.96 | 0.83 |
| **Prestw-911** | 12D02 | (+)-Isoproterenol (+)-bitartrate salt | 0.98 | 0.88 |
| **Prestw-912** | 12D03 | Monobenzone | 0.90 | 0.82 |
| **Prestw-913** | 12D04 | 2-Aminobenzenesulfonamide | 0.92 | 1.12 |
| **Prestw-914** | 12D05 | Estrone | 1.01 | 1.20 |
| **Prestw-915** | 12D06 | Lorglumide sodium salt | 0.83 | 1.16 |
| **Prestw-916** | 12D07 | Nitrendipine | 0.82 | 0.67 |
| **Prestw-917** | 12D08 | Flurbiprofen | 0.82 | 0.87 |
| **Prestw-918** | 12D09 | Nimodipine | 0.87 | 0.81 |

| | | | | |
|---|---|---|---|---|
| **Prestw-919** | 12D10 | Bacitracin | 0.95 | 0.93 |
| **Prestw-920** | 12D11 | L(-)-vesamicol hydrochloride | 0.91 | 0.86 |
| **Prestw-921** | 12E02 | Nizatidine | 0.91 | 0.96 |
| **Prestw-922** | 12E03 | Thioperamide maleate | 0.87 | 0.89 |
| **Prestw-923** | 12E04 | Xamoterol hemifumarate | 0.89 | 0.91 |
| **Prestw-924** | 12E05 | Rolipram | 0.99 | 0.97 |
| **Prestw-925** | 12E06 | Thonzonium bromide | 1.36 | 0.89 |
| **Prestw-926** | 12E07 | Idazoxan hydrochloride | 0.97 | 0.84 |
| **Prestw-927** | 12E08 | Quinapril HCl | 0.92 | 0.83 |
| **Prestw-928** | 12E09 | Nilutamide | 0.91 | 0.90 |
| **Prestw-929** | 12E10 | Ketorolac tromethamine | 0.87 | 0.85 |
| **Prestw-930** | 12E11 | Protriptyline hydrochloride | 0.89 | 0.76 |
| **Prestw-931** | 12F02 | Propofol | 0.87 | 0.94 |
| **Prestw-932** | 12F03 | S(-)Eticlopride hydrochloride | 0.97 | 0.89 |
| **Prestw-933** | 12F04 | Primidone | 0.93 | 0.99 |
| **Prestw-934** | 12F05 | Flucytosine | 0.91 | 1.04 |
| **Prestw-935** | 12F06 | (-)-MK 801 hydrogen maleate | 0.86 | 1.02 |
| **Prestw-936** | 12F07 | Bephenium hydroxynaphthoate | 0.97 | 0.86 |
| **Prestw-937** | 12F08 | Dehydroisoandosterone 3-acetate | 0.90 | 0.90 |
| **Prestw-938** | 12F09 | Benserazide hydrochloride | 0.91 | 0.84 |
| **Prestw-939** | 12F10 | Iodipamide | 0.94 | 0.84 |
| **Prestw-1213** | 12F11 | Allopurinol | 0.95 | 0.81 |
| **Prestw-941** | 12G02 | Pentetic acid | 0.91 | 1.00 |

| | | | | |
|---|---|---|---|---|
| **Prestw-942** | 12G03 | Bretylium tosylate | 0.88 | 0.95 |
| **Prestw-943** | 12G04 | Pralidoxime chloride | 0.85 | 0.93 |
| **Prestw-944** | 12G05 | Phenoxybenzamine hydrochloride | 0.88 | 0.93 |
| **Prestw-945** | 12G06 | Salmeterol | 0.86 | 0.80 |
| **Prestw-946** | 12G07 | Altretamine | 0.88 | 0.83 |
| **Prestw-947** | 12G08 | Prazosin hydrochloride | 0.90 | 0.78 |
| **Prestw-948** | 12G09 | Timolol maleate salt | 0.96 | 0.84 |
| **Prestw-949** | 12G10 | (+,-)-Octopamine hydrochloride | 0.89 | 0.80 |
| **Prestw-1279** | 12G11 | Stavudine | 0.92 | 0.89 |
| **Prestw-951** | 12H02 | Crotamiton | 0.82 | 0.99 |
| **Prestw-1197** | 12H03 | Toremifene | 0.85 | 0.87 |
| **Prestw-536** | 12H04 | (R)-(+)-Atenolol | 0.86 | 1.03 |
| **Prestw-954** | 12H05 | Tyloxapol | 0.88 | 1.03 |
| **Prestw-955** | 12H06 | Florfenicol | 0.87 | 0.95 |
| **Prestw-956** | 12H07 | Megestrol acetate | 0.88 | 1.00 |
| **Prestw-957** | 12H08 | Deoxycorticosterone | 0.83 | 0.93 |
| **Prestw-958** | 12H09 | Urosiol | 0.94 | 0.98 |
| **Prestw-959** | 12H10 | Proparacaine hydrochloride | 0.85 | 1.03 |
| **Prestw-960** | 12H11 | Aminocaproic acid | 0.90 | 0.84 |
| ***Plate 13*** | | | | |
| **Prestw-961** | 13A02 | Denatonium benzoate | 1.01 | 1.02 |
| **Prestw-1259** | 13A03 | Canrenone | 1.04 | 1.03 |
| **Prestw-963** | 13A04 | Enilconazole | 1.02 | 1.08 |

| | | | | |
|---|---|---|---|---|
| **Prestw-964** | 13A05 | Methacycline hydrochloride | 0.98 | 1.03 |
| **Prestw-1415** | 13A06 | Floxuridine | 1.06 | 1.26 |
| **Prestw-966** | 13A07 | Sotalol hydrochloride | 0.98 | 1.26 |
| **Prestw-1267** | 13A08 | Gestrinone | 1.01 | 1.18 |
| **Prestw-968** | 13A09 | Decamethonium bromide | 1.01 | 1.26 |
| **Prestw-969** | 13A10 | 3-Acetamidocoumarin | 0.98 | 1.26 |
| **Prestw-970** | 13A11 | Roxarsone | 0.92 | 1.21 |
| **Prestw-971** | 13B02 | Remoxipride Hydrochloride | 0.94 | 0.95 |
| **Prestw-972** | 13B03 | THIP Hydrochloride | 1.01 | 0.98 |
| **Prestw-973** | 13B04 | Pirlindole mesylate | 0.96 | 0.97 |
| **Prestw-974** | 13B05 | Pronethalol hydrochloride | 0.95 | 1.05 |
| **Prestw-975** | 13B06 | Naftopidil dihydrochloride | 0.96 | 1.06 |
| **Prestw-976** | 13B07 | Tracazolate hydrochloride | 0.97 | 1.04 |
| **Prestw-977** | 13B08 | Zardaverine | 1.02 | 1.19 |
| **Prestw-978** | 13B09 | Memantine Hydrochloride | 0.96 | 1.29 |
| **Prestw-979** | 13B10 | Ozagrel hydrochloride | 1.00 | 1.10 |
| **Prestw-980** | 13B11 | Piribedil hydrochloride | 1.02 | 1.20 |
| **Prestw-981** | 13C02 | Nitrocaramiphen hydrochloride | 0.91 | 0.97 |
| **Prestw-982** | 13C03 | Nandrolone | 1.00 | 1.00 |
| **Prestw-983** | 13C04 | Dimaprit dihydrochloride | 0.99 | 1.08 |
| **Prestw-1459** | 13C05 | Oxfendazol | 1.03 | 1.05 |
| **Prestw-1268** | 13C06 | Guaiacol | 0.92 | 1.19 |
| **Prestw-986** | 13C07 | Proscillaridin A | 0.97 | 0.76 |

| | | | | |
|---|---|---|---|---|
| **Prestw-1316** | 13C08 | Pramipexole | 0.97 | 1.21 |
| **Prestw-1452** | 13C09 | Norgestimate | 1.05 | 1.09 |
| **Prestw-1374** | 13C10 | Chlormadinone acetate | 0.95 | 1.21 |
| **Prestw-1310** | 13C11 | Phenylbutazone | 0.93 | 1.25 |
| **Prestw-991** | 13D02 | Gliquidone | 0.90 | 1.04 |
| **Prestw-992** | 13D03 | Pizotifen malate | 0.92 | 0.82 |
| **Prestw-993** | 13D04 | Ribavirin | 0.95 | 1.10 |
| **Prestw-994** | 13D05 | Cyclopenthiazide | 0.95 | 1.10 |
| **Prestw-995** | 13D06 | Fluvoxamine maleate | 0.90 | 1.21 |
| **Prestw-1321** | 13D07 | Prothionamide | 0.99 | 1.06 |
| **Prestw-997** | 13D08 | Fluticasone propionate | 0.93 | 1.20 |
| **Prestw-998** | 13D09 | Zuclopenthixol hydrochloride | 0.97 | 0.60 |
| **Prestw-999** | 13D10 | Proguanil hydrochloride | 0.87 | 1.14 |
| **Prestw-1000** | 13D11 | Lymecycline | 0.91 | 1.16 |
| **Prestw-1001** | 13E02 | Alfadolone acetate | 0.92 | 1.36 |
| **Prestw-1002** | 13E03 | Alfaxalone | 1.01 | 1.11 |
| **Prestw-1003** | 13E04 | Azapropazone | 0.91 | 1.21 |
| **Prestw-1004** | 13E05 | Meptazinol hydrochloride | 0.93 | 1.17 |
| **Prestw-1005** | 13E06 | Apramycin | 0.97 | 1.25 |
| **Prestw-1006** | 13E07 | Epitiostanol | 0.97 | 1.18 |
| **Prestw-1007** | 13E08 | Fursultiamine Hydrochloride | 0.90 | 1.22 |
| **Prestw-1008** | 13E09 | Gabexate mesilate | 0.96 | 1.08 |
| **Prestw-1009** | 13E10 | Pivampicillin | 1.01 | 1.17 |

| | | | | |
|---|---|---|---|---|
| **Prestw-1010** | 13E11 | Talampicillin hydrochloride | 0.87 | 1.26 |
| **Prestw-1011** | 13F02 | Flucloxacillin sodium | 0.95 | 1.16 |
| **Prestw-1012** | 13F03 | Trapidil | 0.89 | 1.13 |
| **Prestw-1013** | 13F04 | Deptropine citrate | 0.85 | 0.95 |
| **Prestw-1014** | 13F05 | Sertraline | 0.97 | 0.64 |
| **Prestw-1015** | 13F06 | Ethamsylate | 0.99 | 1.28 |
| **Prestw-1016** | 13F07 | Moxonidine | 0.96 | 1.28 |
| **Prestw-1017** | 13F08 | Etilefrine hydrochloride | 1.01 | 1.21 |
| **Prestw-1018** | 13F09 | Alprostadil | 0.93 | 0.86 |
| **Prestw-1019** | 13F10 | Tribenoside | 0.94 | 1.27 |
| **Prestw-1020** | 13F11 | Rimexolone | 0.62 | 1.43 |
| **Prestw-1021** | 13G02 | Isradipine | 0.64 | 0.56 |
| **Prestw-1022** | 13G03 | Tiletamine hydrochloride | 1.00 | 1.06 |
| **Prestw-1023** | 13G04 | Isometheptene mucate | 0.92 | 1.20 |
| **Prestw-1024** | 13G05 | Nifurtimox | 0.94 | 1.16 |
| **Prestw-1025** | 13G06 | Letrozole | 0.87 | 1.20 |
| **Prestw-1026** | 13G07 | Arbutin | 0.84 | 1.21 |
| **Prestw-1027** | 13G08 | Tocainide hydrochloride | 0.94 | 1.37 |
| **Prestw-1028** | 13G09 | Benzathine benzylpenicillin | 0.87 | 1.28 |
| **Prestw-1029** | 13G10 | Risperidone | 0.93 | 1.05 |
| **Prestw-1030** | 13G11 | Torsemide | 0.91 | 1.24 |
| **Prestw-1031** | 13H02 | Halofantrine hydrochloride | 0.90 | 0.87 |
| **Prestw-1032** | 13H03 | Articaine hydrochloride | 0.93 | 0.82 |

| | | | | |
|---|---|---|---|---|
| **Prestw-1033** | 13H04 | Nomegestrol acetate | 0.91 | 0.87 |
| **Prestw-1034** | 13H05 | Pancuronium bromide | 0.89 | 0.91 |
| **Prestw-1035** | 13H06 | Molindone hydrochloride | 0.88 | 1.04 |
| **Prestw-1036** | 13H07 | Alcuronium chloride | 0.88 | 1.10 |
| **Prestw-1037** | 13H08 | Zalcitabine | 0.93 | 1.24 |
| **Prestw-1038** | 13H09 | Methyldopate hydrochloride | 0.91 | 1.27 |
| **Prestw-1039** | 13H10 | Levocabastine hydrochloride | 0.90 | 1.25 |
| **Prestw-1040** | 13H11 | Pyrvinium pamoate | 0.96 | 1.39 |
| ***Plate 14*** | | | | |
| **Prestw-1041** | 14A02 | Etomidate | 1.04 | 0.90 |
| **Prestw-1042** | 14A03 | Tridihexethyl chloride | 1.01 | 0.86 |
| **Prestw-1043** | 14A04 | Penbutolol sulfate | 1.05 | 0.83 |
| **Prestw-1044** | 14A05 | Prednicarbate | 1.05 | 0.81 |
| **Prestw-1045** | 14A06 | Sertaconazole nitrate | 1.01 | 0.82 |
| **Prestw-1046** | 14A07 | Repaglinide | 1.00 | 0.95 |
| **Prestw-1047** | 14A08 | Piretanide | 0.97 | 0.94 |
| **Prestw-1048** | 14A09 | Piperacetazine | 0.86 | 0.84 |
| **Prestw-1049** | 14A10 | Oxyphenbutazone | 0.91 | 1.00 |
| **Prestw-1050** | 14A11 | Quinethazone | 1.32 | 1.24 |
| **Prestw-1051** | 14B02 | Moricizine hydrochloride | 0.96 | 0.93 |
| **Prestw-1052** | 14B03 | Iopanoic acid | 1.00 | 0.76 |
| **Prestw-1053** | 14B04 | Pivmecillinam hydrochloride | 0.97 | 0.87 |
| **Prestw-1054** | 14B05 | Levopropoxyphene napsylate | 0.92 | 0.90 |

| | | | | |
|---|---|---|---|---|
| **Prestw-1055** | 14B06 | Piperidolate hydrochloride | 0.98 | 0.87 |
| **Prestw-1056** | 14B07 | Trifluridine | 0.96 | 0.92 |
| **Prestw-1057** | 14B08 | Oxprenolol hydrochloride | 1.00 | 0.92 |
| **Prestw-1058** | 14B09 | Ondansetron Hydrochloride | 0.96 | 0.91 |
| **Prestw-1059** | 14B10 | Propoxycaine hydrochloride | 1.03 | 0.90 |
| **Prestw-1060** | 14B11 | Oxaprozin | 0.95 | 0.94 |
| **Prestw-1061** | 14C02 | Phensuximide | 1.02 | 0.84 |
| **Prestw-1062** | 14C03 | Ioxaglic acid | 1.07 | 1.10 |
| **Prestw-1063** | 14C04 | Naftifine hydrochloride | 0.97 | 0.84 |
| **Prestw-1064** | 14C05 | Meprylcaine hydrochloride | 0.90 | 0.94 |
| **Prestw-1065** | 14C06 | Milrinone | 0.97 | 0.99 |
| **Prestw-1066** | 14C07 | Methantheline bromide | 1.00 | 0.91 |
| **Prestw-1067** | 14C08 | Ticarcillin sodium | 0.96 | 1.02 |
| **Prestw-1068** | 14C09 | Thiethylperazine malate | 1.05 | 0.57 |
| **Prestw-1069** | 14C10 | Mesalamine | 1.00 | 1.00 |
| **Prestw-1362** | 14C11 | Vorinostat | 1.04 | 1.00 |
| **Prestw-1071** | 14D02 | Imidurea | 0.89 | 0.96 |
| **Prestw-1072** | 14D03 | Lansoprazole | 0.94 | 0.90 |
| **Prestw-1073** | 14D04 | Bethanechol chloride | 0.97 | 0.86 |
| **Prestw-1074** | 14D05 | Cyproterone acetate | 1.01 | 0.95 |
| **Prestw-1075** | 14D06 | (R)-Propranolol hydrochloride | 0.95 | 1.08 |
| **Prestw-1076** | 14D07 | Ciprofibrate | 1.03 | 1.05 |
| **Prestw-1420** | 14D08 | Formestane | 0.98 | 0.97 |

| | | | | |
|---|---|---|---|---|
| **Prestw-1078** | 14D09 | Benzylpenicillin sodium | 0.94 | 0.97 |
| **Prestw-1079** | 14D10 | Chlorambucil | 0.94 | 1.14 |
| **Prestw-1080** | 14D11 | Methiazole | 0.95 | 1.06 |
| **Prestw-1081** | 14E02 | (S)-propranolol hydrochloride | 0.93 | 1.06 |
| **Prestw-1082** | 14E03 | (-)-Eseroline fumarate salt | 0.94 | 1.02 |
| **Prestw-1294** | 14E04 | Isosorbide mononitrate | 0.94 | 1.03 |
| **Prestw-1084** | 14E05 | Leucomisine | 0.98 | 1.07 |
| **Prestw-1493** | 14E06 | Topiramate | 0.96 | 1.05 |
| **Prestw-1086** | 14E07 | D-cycloserine | 0.95 | 1.06 |
| **Prestw-1087** | 14E08 | 2-Chloropyrazine | 1.02 | 1.07 |
| **Prestw-1088** | 14E09 | (+,-)-Synephrine | 0.98 | 1.11 |
| **Prestw-1089** | 14E10 | (S)-(-)-Cycloserine | 1.00 | 1.03 |
| **Prestw-1090** | 14E11 | Homosalate | 0.94 | 1.13 |
| **Prestw-1091** | 14F02 | Spaglumic acid | 0.95 | 1.10 |
| **Prestw-1092** | 14F03 | Ranolazine | 0.93 | 1.06 |
| **Prestw-1443** | 14F04 | Misoprostol | 0.94 | 1.18 |
| **Prestw-1094** | 14F05 | Sulfadoxine | 0.95 | 1.01 |
| **Prestw-1095** | 14F06 | Cyclopentolate hydrochloride | 0.97 | 1.10 |
| **Prestw-1096** | 14F07 | Estriol | 0.94 | 1.10 |
| **Prestw-1097** | 14F08 | (-)-Isoproterenol hydrochloride | 0.95 | 1.13 |
| **Prestw-1339** | 14F09 | Sarafloxacin | 0.99 | 1.13 |
| **Prestw-1099** | 14F10 | Nialamide | 1.01 | 1.09 |
| **Prestw-1195** | 14F11 | Toltrazuril | 0.96 | 1.02 |

| | | | | |
|---|---|---|---|---|
| **Prestw-1101** | 14G02 | Perindopril | 0.88 | 1.08 |
| **Prestw-1102** | 14G03 | Fexofenadine HCl | 0.90 | 0.99 |
| **Prestw-1202** | 14G04 | 4-aminosalicylic acid | 0.90 | 1.15 |
| **Prestw-1104** | 14G05 | Clonixin Lysinate | 0.96 | 1.04 |
| **Prestw-1105** | 14G06 | Verteporfin | 0.84 | 1.12 |
| **Prestw-1106** | 14G07 | Meropenem | 0.95 | 0.92 |
| **Prestw-1107** | 14G08 | Ramipril | 0.92 | 1.03 |
| **Prestw-1108** | 14G09 | Mephenytoin | 0.94 | 0.96 |
| **Prestw-1109** | 14G10 | Rifabutin | 0.89 | 0.84 |
| **Prestw-1110** | 14G11 | Parbendazole | 0.89 | 1.09 |
| **Prestw-1111** | 14H02 | Mecamylamine hydrochloride | 0.92 | 0.81 |
| **Prestw-1112** | 14H03 | Procarbazine hydrochloride | 0.96 | 0.81 |
| **Prestw-1113** | 14H04 | Viomycin sulfate | 0.87 | 0.85 |
| **Prestw-1114** | 14H05 | Saquinavir mesylate | 0.92 | 0.78 |
| **Prestw-1115** | 14H06 | Ronidazole | 0.96 | 0.99 |
| **Prestw-1116** | 14H07 | Dorzolamide hydrochloride | 0.89 | 1.14 |
| **Prestw-1117** | 14H08 | Azaperone | 0.96 | 1.22 |
| **Prestw-1118** | 14H09 | Cefepime hydrochloride | 0.92 | 1.23 |
| **Prestw-1119** | 14H10 | Clocortolone pivalate | 0.90 | 1.23 |
| **Prestw-1120** | 14H11 | Nadifloxacin | 1.07 | 1.20 |
| ***Plate 15*** | | | | |
| **Prestw-1283** | 15A02 | Buspirone hydrochloride | 1.07 | 0.90 |
| **Prestw-1222** | 15A03 | Anastrozole | 1.36 | 1.09 |

| | | | | |
|---|---|---|---|---|
| **Prestw-1399** | 15A04 | Doxycycline hydrochloride | 1.30 | 0.78 |
| **Prestw-1345** | 15A05 | Sulbactam | 1.11 | 0.85 |
| **Prestw-1414** | 15A06 | Fleroxacin | 1.08 | 0.87 |
| **Prestw-1315** | 15A07 | Potassium clavulanate | 1.04 | 1.08 |
| **Prestw-1482** | 15A08 | Valproic acid | 1.08 | 1.16 |
| **Prestw-1280** | 15A09 | Mepivacaine hydrochloride | 1.06 | 1.06 |
| **Prestw-1478** | 15A10 | Rifaximin | 1.09 | 1.06 |
| **Prestw-1473** | 15A11 | Estradiol Valerate | 1.01 | 1.16 |
| **Prestw-1206** | 15B02 | Acetylcysteine | 1.03 | 0.86 |
| **Prestw-1435** | 15B03 | Melengestrol acetate | 1.43 | 0.80 |
| **Prestw-1246** | 15B04 | Bromhexine hydrochloride | 1.08 | 0.73 |
| **Prestw-1223** | 15B05 | Anethole-trithione | 1.03 | 0.80 |
| **Prestw-1476** | 15B06 | Amcinonide | 1.08 | 0.72 |
| **Prestw-1256** | 15B07 | Caffeine | 1.08 | 0.76 |
| **Prestw-1262** | 15B08 | Carvedilol | 1.17 | 0.73 |
| **Prestw-1282** | 15B09 | Methenamine | 1.06 | 0.99 |
| **Prestw-1308** | 15B10 | Phentermine hydrochloride | 1.11 | 0.98 |
| **Prestw-1394** | 15B11 | Diclazuril | 1.12 | 0.92 |
| **Prestw-1249** | 15C02 | Famciclovir | 1.09 | 0.84 |
| **Prestw-1398** | 15C03 | Dopamine hydrochloride | 1.06 | 1.05 |
| **Prestw-1263** | 15C04 | Cefdinir | 1.04 | 0.78 |
| **Prestw-1261** | 15C05 | Carprofen | 1.13 | 0.91 |
| **Prestw-1371** | 15C06 | Celecoxib | 1.06 | 0.87 |

| | | | | |
|---|---|---|---|---|
| **Prestw-1258** | 15C07 | Candesartan | 1.04 | 0.83 |
| **Prestw-1483** | 15C08 | Fludarabine | 1.03 | 0.90 |
| **Prestw-1484** | 15C09 | Cladribine | 0.98 | 1.12 |
| **Prestw-1356** | 15C10 | Vardenafil | 1.07 | 1.00 |
| **Prestw-1417** | 15C11 | Fluconazole | 0.98 | 0.84 |
| **Prestw-1203** | 15D02 | 5-fluorouracil | 1.01 | 0.95 |
| **Prestw-1487** | 15D03 | Mesna | 0.95 | 1.06 |
| **Prestw-1444** | 15D04 | Mitotane | 0.99 | 0.83 |
| **Prestw-1497** | 15D05 | Ambrisentan | 0.93 | 0.95 |
| **Prestw-1479** | 15D06 | Triclosan | 1.06 | 0.91 |
| **Prestw-1401** | 15D07 | Enoxacin | 1.06 | 0.89 |
| **Prestw-1307** | 15D08 | Olopatadine hydrochloride | 1.08 | 0.85 |
| **Prestw-1187** | 15D09 | Granisetron | 1.03 | 1.00 |
| **Prestw-1224** | 15D10 | Anthralin | 1.04 | 1.07 |
| **Prestw-1492** | 15D11 | Lamotrigine | 0.99 | 0.76 |
| **Prestw-1383** | 15E02 | Clofibrate | 0.93 | 1.09 |
| **Prestw-1481** | 15E03 | Cyclophosphamide | 0.91 | 1.05 |
| **Prestw-1229** | 15E04 | Aripiprazole | 1.03 | 1.01 |
| **Prestw-1405** | 15E05 | Ethinylestradiol | 1.03 | 0.97 |
| **Prestw-1419** | 15E06 | Fluocinolone acetonide | 0.93 | 0.90 |
| **Prestw-1343** | 15E07 | Sparfloxacin | 0.95 | 0.90 |
| **Prestw-1390** | 15E08 | Desloratadine | 0.98 | 0.51 |
| **Prestw-1378** | 15E09 | Clarithromycin | 0.92 | 0.94 |

| | | | | |
|---|---|---|---|---|
| **Prestw-1199** | 15E10 | Tripelennamine hydrochloride | 0.98 | 0.92 |
| **Prestw-1352** | 15E11 | Tulobuterol | 1.05 | 1.00 |
| **Prestw-1196** | 15F02 | Topotecan | 0.99 | 1.09 |
| **Prestw-1232** | 15F03 | Atorvastatin | 1.01 | 1.03 |
| **Prestw-1234** | 15F04 | Azithromycin | 0.96 | 0.98 |
| **Prestw-1286** | 15F05 | Ibudilast | 0.92 | 0.94 |
| **Prestw-1433** | 15F06 | Losartan | 0.87 | 0.93 |
| **Prestw-1236** | 15F07 | Benztropine mesylate | 0.91 | 0.61 |
| **Prestw-1359** | 15F08 | Vecuronium bromide | 1.00 | 0.88 |
| **Prestw-1350** | 15F09 | Telmisartan | 1.05 | 1.02 |
| **Prestw-1490** | 15F10 | Nalmefene hydrochloride | 0.97 | 0.85 |
| **Prestw-1241** | 15F11 | Bifonazole | 0.85 | 0.87 |
| **Prestw-1265** | 15G02 | Gatifloxacin | 0.95 | 0.99 |
| **Prestw-1244** | 15G03 | Bosentan | 1.07 | 0.93 |
| **Prestw-1266** | 15G04 | Gemcitabine | 0.79 | 0.84 |
| **Prestw-1190** | 15G05 | Olmesartan | 0.84 | 0.59 |
| **Prestw-1480** | 15G06 | Racepinephrine HCl | 0.91 | 0.94 |
| **Prestw-1189** | 15G07 | Montelukast | 0.95 | 1.02 |
| **Prestw-1180** | 15G08 | Docetaxel | 0.94 | 0.91 |
| **Prestw-1376** | 15G09 | Cilnidipine | 0.96 | 0.97 |
| **Prestw-1291** | 15G10 | Imiquimod | 0.97 | 1.00 |
| **Prestw-1423** | 15G11 | Fosinopril | 0.87 | 0.97 |
| **Prestw-1290** | 15H02 | Imatinib | 0.90 | 0.72 |

| | | | | |
|---|---|---|---|---|
| **Prestw-1446** | 15H03 | Moxifloxacin | 0.89 | 0.70 |
| **Prestw-1421** | 15H04 | Formoterol fumarate | 0.91 | 0.65 |
| **Prestw-1338** | 15H05 | Rufloxacin | 0.94 | 0.77 |
| **Prestw-1319** | 15H06 | Pravastatin | 0.99 | 0.82 |
| **Prestw-1337** | 15H07 | Rosiglitazone | 0.90 | 0.98 |
| **Prestw-1334** | 15H08 | Rivastigmine | 0.90 | 0.98 |
| **Prestw-1342** | 15H09 | Sildenafil | 0.92 | 1.03 |
| **Prestw-1207** | 15H10 | Acetylsalicylic acid | 0.88 | 0.99 |
| **Prestw-1472** | 15H11 | Hexachlorophene | 0.95 | 0.99 |

**Supplemental Table 2. Compounds of the Prestwick Chemical Library excluded from analysis.** Compounds that had a fold-change ≥ 1.3 in the K562-NL alone condition were excluded from analysis as these drugs cytotoxic to K562-NL cells in absence of NK92 cells.

| Prestw number | Plate # / Well position | Chemical name | average fold-change |
|---|---|---|---|
| **Prestw-349** | 05C10 | Ritodrine hydrochloride | 1.69 |
| **Prestw-369** | 05E10 | Meloxicam | 1.50 |
| **Prestw-339** | 05B10 | Guanfacine hydrochloride | 1.44 |
| **Prestw-925** | 12E06 | Thonzonium bromide | 1.36 |
| **Prestw-143** | 02G04 | Chlorhexidine | 1.36 |
| **Prestw-1152** | 05F10 | Nefazodone HCl | 1.34 |
| **Prestw-1435** | 15B03 | Melengestrol acetate | 1.43 |
| **Prestw-705** | 09G06 | Methyl benzethonium chloride | 1.32 |
| **Prestw-1050** | 14A11 | Quinethazone | 1.32 |
| **Prestw-1399** | 15A04 | Doxycycline hydrochloride | 1.30 |
| **Prestw-777** | 10F08 | Alexidine dihydrochloride | 16.01 |
| **Prestw-1222** | 15A03 | Anastrozole | 1.36 |

**Supplemental Table 3. Z' factor for individual assay plates from the screening of the Prestwick Chemical Library.** Z' factor was calculated using the positive and negative control luminescent values from each plate.

| Plate # | K562-NL alone | 1:1 E:T |
|---|---|---|
| **1** | 0.84 | 0.64 |
| **2** | 0.67 | 0.52 |
| **3** | 0.63 | 0.20 |
| **4** | 0.75 | 0.43 |
| **5** | 0.66 | 0.50 |
| **6** | 0.64 | 0.56 |
| **7** | 0.74 | 0.80 |
| **8** | 0.73 | 0.28 |
| **9** | 0.75 | 0.54 |
| **10** | 0.81 | 0.55 |
| **11** | 0.80 | 0.56 |
| **12** | 0.79 | 0.68 |
| **13** | 0.77 | 0.51 |
| **14** | 0.74 | -0.06 |
| **15** | 0.50 | 0.06 |
| **Average** | 0.72 | 0.44 |

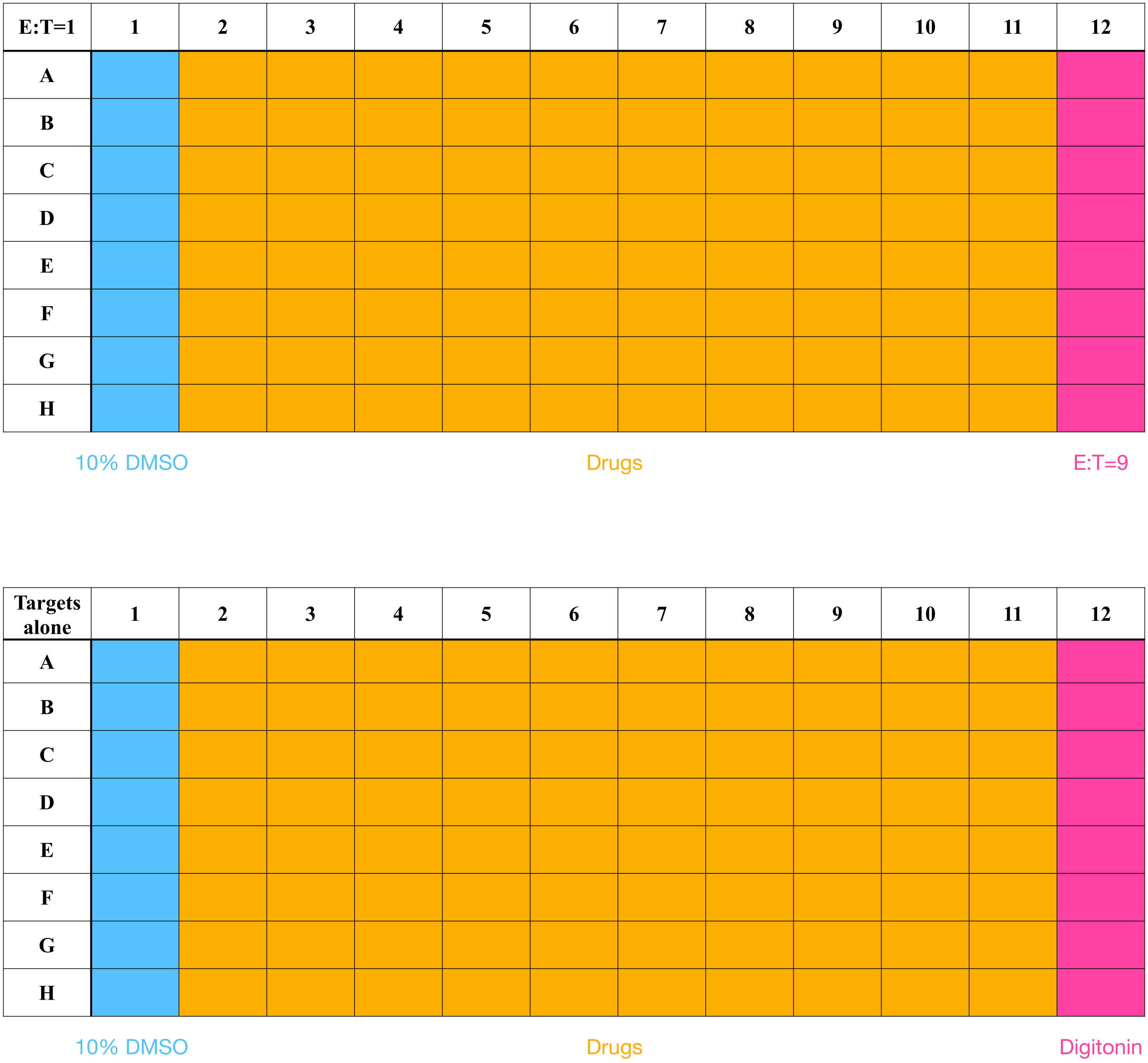


Supplementary Figure 1

A

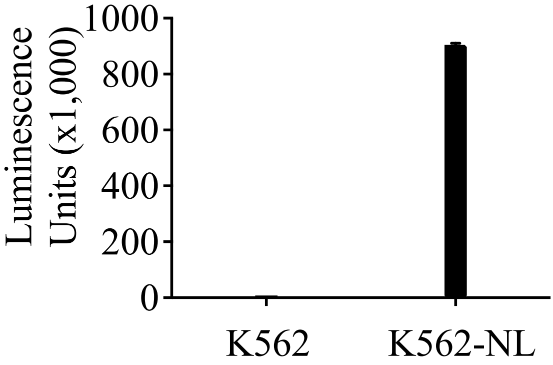


B

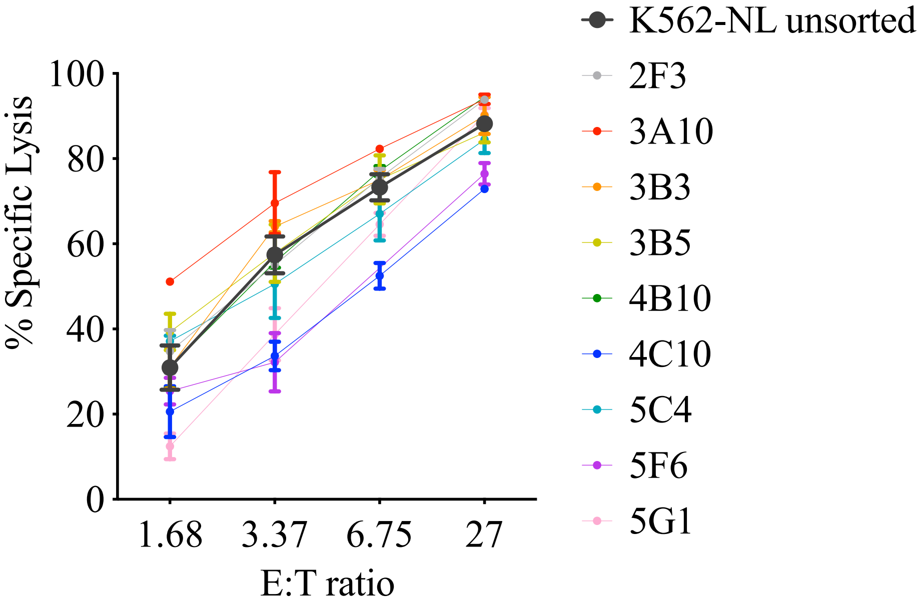


Supplementary Figure 2

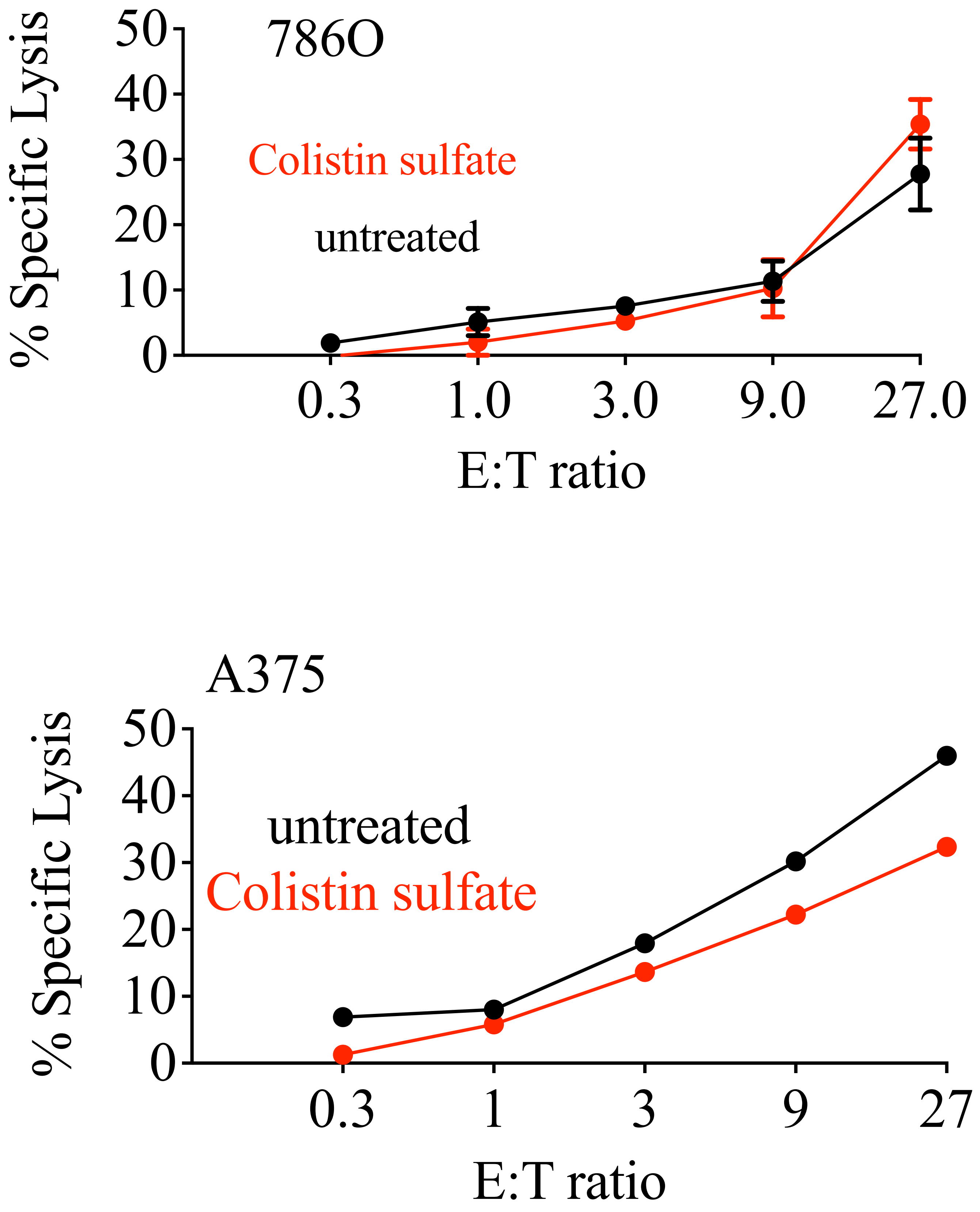


Supplementary Figure 3

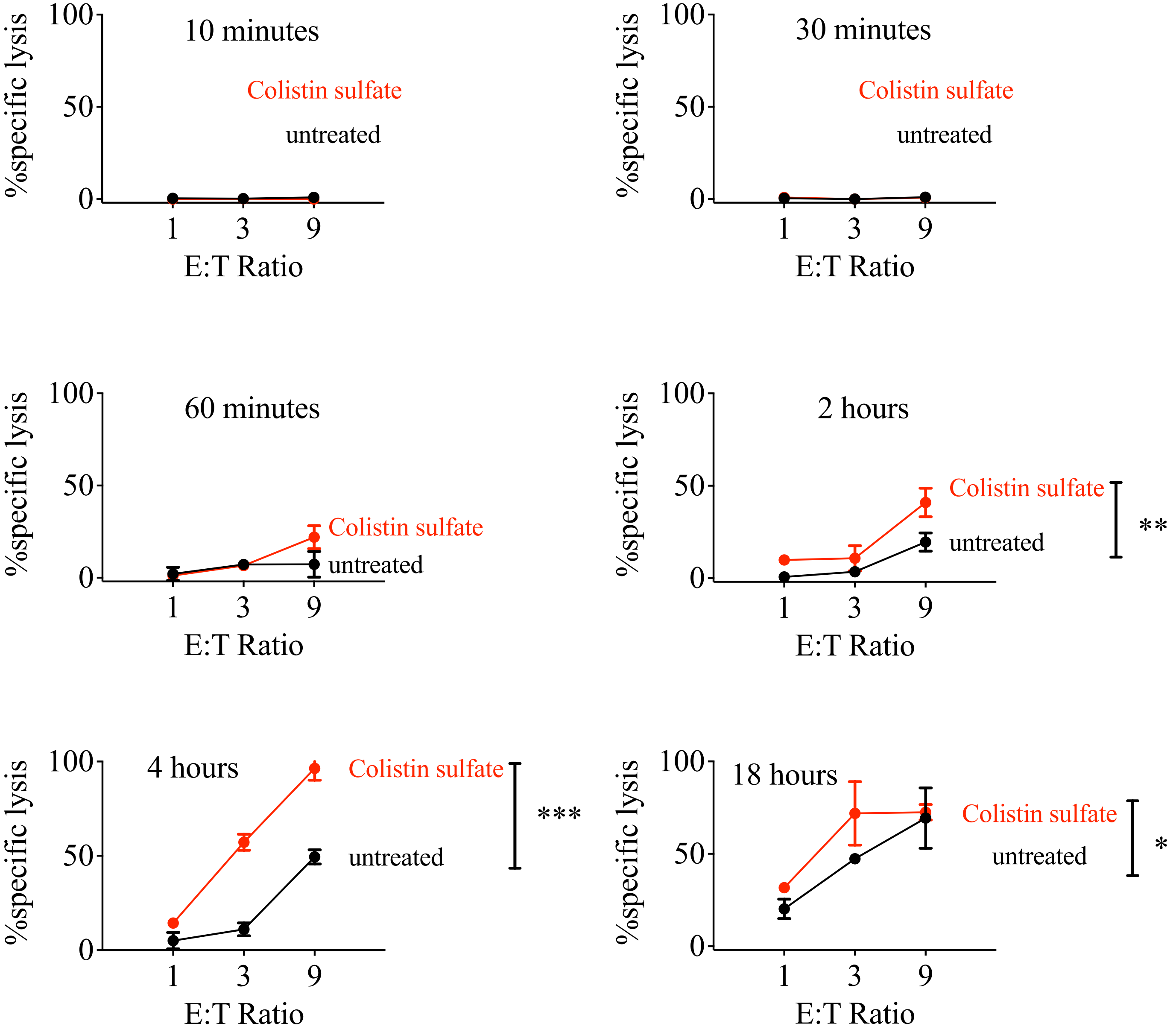


Supplementary Figure 4
